# Neurotransmitter deficits from frontotemporal lobar degeneration

**DOI:** 10.1093/brain/awx327

**Published:** 2018-01-24

**Authors:** Alexander G Murley, James B Rowe

**Affiliations:** 1 Department of Clinical Neurosciences, University of Cambridge, Herchel Smith Building, Robinson Way, Cambridge, CB2 0SZ, UK; 2 MRC Cognition and Brain Sciences Unit, University of Cambridge, 15 Chaucer Road, Cambridge, CB2 7EF, UK; 3 Behavioural and Clinical Neurosciences Institute, University of Cambridge, Sir William Hardy Building, Downing Street, Cambridge, CB2 3EB, UK

**Keywords:** frontotemporal dementia, progressive supranuclear palsy, corticobasal degeneration, neurotransmitters, dementia

## Abstract

Frontotemporal lobar degeneration causes a spectrum of complex degenerative disorders including frontotemporal dementia, progressive supranuclear palsy and corticobasal syndrome, each of which is associated with changes in the principal neurotransmitter systems. We review the evidence for these neurochemical changes and propose that they contribute to symptomatology of frontotemporal lobar degeneration, over and above neuronal loss and atrophy. Despite the development of disease-modifying therapies, aiming to slow neuropathological progression, it remains important to advance symptomatic treatments to reduce the disease burden and improve patients’ and carers’ quality of life. We propose that targeting the selective deficiencies in neurotransmitter systems, including dopamine, noradrenaline, serotonin, acetylcholine, glutamate and gamma-aminobutyric acid is an important strategy towards this goal. We summarize the current evidence-base for pharmacological treatments and suggest strategies to improve the development of new, effective pharmacological treatments.

## Teaser

Murley and Rowe review the neurochemical changes arising from frontotemporal lobar degeneration, including the syndromes frontotemporal dementia, progressive supranuclear palsy and corticobasal degeneration. The evidence base from *in vivo* and post-mortem human studies, and preclinical models, suggests new strategies to facilitate the development of symptomatic pharmacological treatments, in stratified populations.

## Introduction

Frontotemporal lobar degeneration (FTLD) causes diverse clinical syndromes, including frontotemporal dementia (FTD), progressive supranuclear palsy (PSP) and corticobasal syndrome (CBS) (MacKenzie *et al.*, 2010; Riedl *et al.*, 2014). In recent years there has been marked progress in defining these syndromes in terms of their clinical diagnostic criteria (Gorno-Tempini *et al.*, 2011; Rascovsky *et al.*, 2011; Armstrong *et al.*, 2013; Höglinger *et al.*, 2017), genetic association (Seelaar *et al.*, 2011; Baizabal-Carvallo and Jankovic, 2016), pathology (MacKenzie *et al.*, 2010), and clinical and imaging biomarkers (Whitwell *et al.*, 2005; Hughes *et al.*, 2013; Skillback *et al.*, 2014; Rohrer *et al.*, 2015*b*; Ranasinghe *et al.*, 2016). These advances have supported the development of candidate disease-modifying therapeutics (Boxer and Boeve, 2007; Tsai and Boxer, 2014; Stamelou and Höglinger, 2016). However, treatments that slow or halt disease progression after symptoms begin must be accompanied by more effective treatment of symptoms to reduce the overall burden of disease. One strategy is to reverse neurotransmitter deficits, similar to dopaminergic therapy of Parkinson’s disease or cholinergic therapy for Alzheimer’s disease. Novel symptomatic drug treatment would improve patients’ and their families’ quality of life.

Recent changes in the clinical and pathological characterization of the major clinical syndromes caused by FTLD give anatomical and pharmacological insights that call for a reappraisal of the neurotransmitter literature. We adopt the clinical labels as set out in current consensus diagnostic criteria for the behavioural variant FTD (bvFTD) (Rascovsky *et al.*, 2011), semantic variant of primary progressive aphasia (svPPA) (Gorno-Tempini *et al.*, 2011), logopenic variant of PPA (lvPPA) (Gorno-Tempini *et al.*, 2011), non-fluent agrammatic variant PPA (nfvPPA) (Gorno-Tempini *et al.*, 2011), CBS (Armstrong *et al.*, 2013) and PSP (Höglinger *et al.*, 2017). However, older studies may have used different terms or overlooked the evolution of phenotype that obscures the boundaries between groups as the disease progresses (Coyle-Gilchrist *et al.*, 2016). Where these changes are relevant to the interpretation of neurotransmitter effects, we make variations from the current standard classification explicit, but otherwise consider semantic dementia as semantic variant PPA and progressive non-fluent aphasia as non-fluent agrammatic variant PPA.

Here we review the pharmacological abnormalities associated with FTLD in terms of regional changes in neurotransmitter synthesis, release, reuptake, catabolism, and synaptic binding. We focus on the major neurotransmitter systems, dopamine, noradrenaline, serotonin, acetylcholine, glutamate and gamma aminobutyric acid (GABA) both individually (including their receptor subtypes) and the interactions between them. Table 1 provides a summary of the available evidence, with full information on references by disease and by neurotransmitter in Supplementary Table 1.

**Table 1:** Summary of neurotransmitter deficits in FTLD

Neurotransmitter pathway	FTD	PSP	CBS
**Dopamine**			
Dopaminergic neurons	↓↓	↓↓	↓↓
Dopamine receptors	↓	↓↓^a^	↔
**Noradrenaline**			
Noradrenergic neurons	↔	↓↓	na
Noradrenergic receptors	na	na	na
**Serotonin**			
Serotonergic neurons	↓↓	↓	↓
Serotonergic receptors	↓↓	↑	na
**Acetylcholine**			
Cholinergic neurons	↔^b^	↓↓	↓↓
Cholinergic receptors	↔/↓	↔/↓	na
**Glutamate**			
Glutamatergic neurons	↓↓	↓↓	na
Glutamatergic receptors	↓↓	↔	na
**GABA**			na
GABAergic neurons	↓	↓↓	na
GABA receptors	na	↓	na

A more detailed table, including references, is included as Supplementary Fig. 1.

↓↓ = moderate/severe deficit; ↓ = mild deficit; ↑/↔/↓ = conflicting or inconsistent results; ↔ = no significant change; ↑ = mild increase; na = no available evidence.

^a^In PSP D2 receptors are reduced in the striatum and basal ganglia but D1 receptors appear to be preserved.

^b^Cholinergic neurons are reduced in the nucleus basalis but are preserved in the cerebral cortex in bvFTD. In nfvPPA there is greater evidence of a cholinergic deficit with atrophy of basal forebrain cholinergic nuclei.

## Dopamine

**Figure 1:**
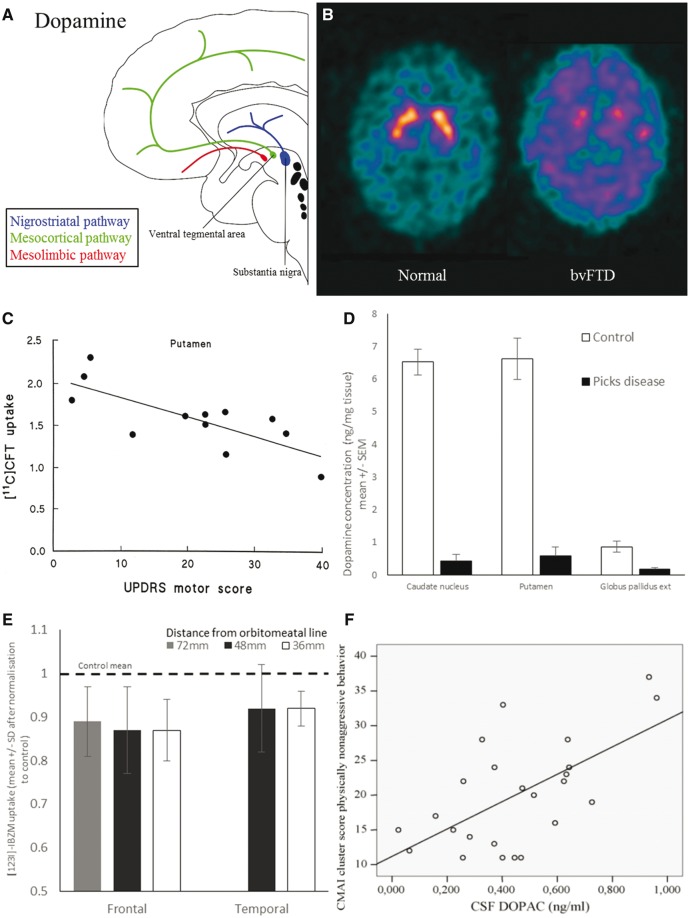
**Dopamine deficits in FTD.** (**A**) Schematic illustration of dopaminergic pathways. (**B**) Ioflupane SPECT scan showing loss of pre-synaptic dopaminergic neurons in the striatum of FTD compared with normal scan. (**C**) Loss of dopaminergic neurons in the putamen (measured by ^11^C-CFT-PET) correlates with severity of extra-pyramidal motor symptoms (Unified Parkinson’s Disease Rating Scale motor score). From Rinne *et al.* (2002). Reprinted with permission from Wolter Kluwer. (**D**) Dopamine levels are reduced in the caudate, putamen and globus pallidus. Graph of data from Kanazawa *et al.* (1988). Reprinted with permission from Elsevier. (**E**) There is loss of D2 dopamine receptors in the frontal lobes (as measured by ^123^I-IBZM-PET). Graph of data from Frisoni *et al.* (1994). Reprinted with permission from Elsevier. (**F**) CSF DOPAC levels (3,4-dihydroxyphenylacetic acid, a dopamine metabolite) correlate with behavioural disturbance. From Engelborghs *et al.* (2008). Reprinted with permission from Elsevier.

Dopaminergic deficits are widely associated with Parkinson’s disease but are also a common feature of FTLD. The majority of dopaminergic neurons originate in the ventral midbrain and form nigrostriatal, mesolimbic and mesocortical projections (Fig. 1A). Nigrostriatal neurons from the substantia nigra pars compacta terminate in the striatum, regulating cortico-striato-thalamo-cortical loops for motor, oculomotor and cognitive control (Rowe and Rittman, 2016). The motor circuit regulates movement, both in facilitating (via the direct pathway) and inhibiting (via the indirect pathway) actions. Loss of dopaminergic neurons in the nigrostriatal pathway causes parkinsonism in Parkinson’s disease, but also in FTLD. Additional mesolimbic and mesocortical dopaminergic neurons from the ventral tegmental area regulate reward, learning and motivation-related behaviour (Wise, 2004). The mesolimbic tract projects principally to the nucleus accumbens in the striatum and to the amygdala and hippocampus, affecting motivation, hedonia and reward (incentive salience). Changes to the mesolimbic tract may also exacerbate compulsion and impulsivity. The mesocortical tract (which projects to the prefrontal, cingulate and perirhinal cortices) regulates motivation, emotion, reward and desire, including learning of the value of goal-directed actions. Dopamine binds to five types of G protein coupled receptors; D_1_-class (D_1_ and D_5_) and D_2_-class (D_2,_ D_3_ and D_4_), which differ in their response to dopamine agonists and antagonists (Beaulieu and Gainetdinov, 2011; Southan *et al.*, 2016). The different receptor subtypes have distinct distribution densities across brain regions and are associated with different, although overlapping, effects on cognition and movement (Beaulieu and Gainetdinov, 2011), and may be differentially affected by FTLD.

### Frontotemporal dementia

There is clinical and experimental evidence of a nigrostriatal deficit in many cases of FTD, with loss of pre-synaptic dopaminergic neurons, reduced dopamine levels, reduced dopamine transporter binding, and abnormal dopamine receptor binding. Extrapyramidal symptoms of bradykinesia, rigidity and gait dysfunction are seen in up to 70% of patients at some stage during the disease course (Rinne *et al.*, 2002; Padovani *et al.*, 2007; Kertesz *et al.*, 2011; Gil-Navarro *et al.*, 2013). *In vivo* imaging reveals that dopamine transporter levels (a marker of presynaptic neuron integrity in the striatum) are reduced in the caudate and putamen (Fig. 1B) (Rinne *et al.*, 2002; Sedaghat *et al.*, 2007). The degree of this loss correlates with extra-pyramidal symptom severity (Fig. 1C) (Rinne *et al.*, 2002; Sedaghat *et al.*, 2007).

In bvFTD there are low levels of dopamine, measured by high performance liquid chromatography, in the putamen, caudate and substantia nigra (Kanazawa *et al.*, 1988; Nagaoka *et al.*, 1995) (Fig. 1D). Parkinsonism is commonly seen in bvFTD, especially when caused by certain genetic mutations (Baizabal-Carvallo and Jankovic, 2016). Mutations on chromosome 17, including in the *MAPT* (Hutton *et al.*, 1998) and *PGRN* (Baker *et al.*, 2006) genes, are associated with rigidity, akinesia and neuronal loss in the substantia nigra, although symptom onset and severity vary with each specific mutation (Foster *et al.*, 1997; Pickering-Brown *et al.*, 2002; Le Ber *et al.*, 2008; Siuda *et al.*, 2014; Baizabal-Carvallo and Jankovic, 2016). For example, an early PET study in three patients with FTD associated with a chromosome 17 mutation found severe reduction in presynaptic dopaminergic neurons with normal D2 receptor levels in the striatum (Pal *et al.*, 2001). The hexanucleatide expansion in the *C9orf72* gene on chromosome 9 is most typically associated with FTD with amyotrophic lateral sclerosis (Rohrer *et al.*, 2015*a*), but up to half of patients have parkinsonism, with decreased dopamine transporter levels in the basal ganglia (Boeve *et al.*, 2012; O’Dowd *et al.*, 2012). Extra-pyramidal symptoms are also seen with mutations in *CHMP2B*, *FUS*, *TARDBP*, *TREM2* and *VCP* (Siuda *et al.*, 2014; Baizabal-Carvallo and Jankovic, 2016). In non-fluent agrammatic variant PPA, there is frequent loss of dopaminergic neurons in the striatum (Gil-Navarro *et al.*, 2013), which underlies the frequent progression of motor symptoms in this disorder, and its clinical overlap with CBS and PSP (Rohrer *et al.*, 2010). Parkinsonism in bvFTD and non-fluent agrammatic variant PPA appears to occur with all types of underlying pathology; tau (Hutton *et al.*, 1998), TDP-43 (Boeve *et al.*, 2012) and FUS pathology (Deng *et al.*, 2014) are all associated with motor symptoms (Baizabal-Carvallo and Jankovic, 2016).

In addition to extrapyramidal motor features, degeneration of dopaminergic tracts, especially the mesocortical pathway, could contribute to behavioural symptoms of FTD. For example, D2 dopamine receptors are reduced in the frontal lobes of patients with FTD (Frisoni *et al.*, 1994) (Fig. 1E), while CSF levels of dopamine and its metabolites are reduced in some (Sjogren *et al.*, 1998) but not all studies (Vermeiren *et al.*, 2013). CSF levels of dopamine correlate with agitation and caregiver burden in FTD (Fig. 1F) (Engelborghs *et al.*, 2008). However, these findings contrast with a study that found higher dopamine levels in the prefrontal cortex at post-mortem (Vermeiren *et al.*, 2016). Such inconsistencies may result from technological or methodological differences in tissue preparation or analysis, but they may also reflect true heterogeneity in the FTD population, especially in small post-mortem analyses.

Aggression, agitation and psychosis are distressing and burdensome aspects of FTD. Antipsychotic medications with dopaminergic receptor affinity are often used to treat them but patients can be extremely sensitive to the extra-pyramidal side effects due to pretreatment nigrostriatal deficits (Pijnenburg *et al.*, 2003). Atypical antipsychotics such as quetiapine, olanzapine or clozapine cause fewer extrapyramidal side effects (Moretti *et al.*, 2003*b*) while noting that there is less evidence for their efficacy in dementia. In an open label, non-randomized study, olanzapine improved behavioural fluctuations, wandering and irritability (Moretti *et al.*, 2003*b*). An alternative strategy using methylphenidate, a noradrenaline and dopamine reuptake inhibitor, reduced risk-taking behaviour in a small double-blind, placebo-controlled study, but without effects on a wide range of cognitive tasks (Rahman *et al.*, 2006). There is a case report of improved behaviour with methylphenidate and bupropion (another noradrenaline and dopamine reuptake inhibitor) in one patient with FTD (Goforth *et al.*, 2004). In addition to the uncertainty over dopaminergic strategies to treat cognitive and behavioural symptoms in FTD, systematic evidence is lacking of the efficacy of levodopa or dopamine agonists to ameliorate parkinsonism in FTD, with only case reports of benefit in some patients (Chow, 2002; Tsai and Boxer, 2014).

### Progressive supranuclear palsy

**Figure 2:**
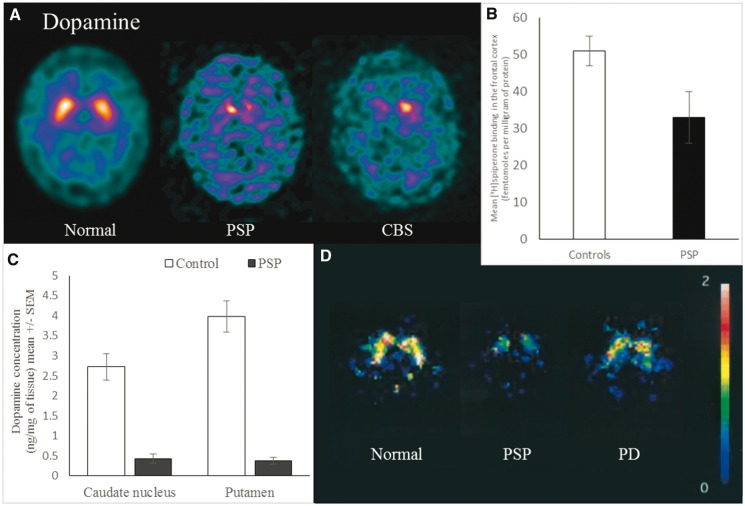
**Dopamine deficits in PSP and CBS.** (**A**) Ioflupane SPECT scan showing reduced pre-synaptic dopaminergic neurons in the striatum of PSP and CBS compared to a normal scan. (**B**) Post-mortem dopamine receptor levels (measured by spiperone binding) are reduced in the frontal cortex in PSP. Graph of data from Ruberg *et al.* (1985). Reprinted with permission from Wiley. (**C**) Dopamine levels are reduced in the caudate nucleus and putamen in PSP. Graph of data from Ruberg *et al.* (1985). (**D**) D2 dopamine receptor levels (measured by ^123^I-iodobenzofuran SPECT) are reduced in the striatum of PSP when compared with healthy controls and Parkinson’s disease. From Oyanagi (2002). Reprinted with permission from Wiley.

*In vivo* and post-mortem studies show that the extrapyramidal features of PSP are associated with a severe loss of dopaminergic neurons and changes in dopamine receptors, particularly D2 receptors. Pathological tau aggregates, including neuronal tangles and glial inclusions, develop in areas with a high density of dopaminergic neurons including the substantia nigra and striatum (Litvan *et al.*, 1996; Hardman *et al.*, 1997). There is marked loss of pigmented dopaminergic neurons in the substantia nigra pars compacta on examination post-mortem (Hardman *et al.*, 1997; Oyanagi *et al.*, 2001). There is also loss of both dopaminergic neurons and dopamine receptors in the striatum (Baron *et al.*, 1986; Kim *et al.*, 2002; Oyanagi, 2002; Im *et al.*, 2006; Oh *et al.*, 2012). Dopamine transporter binding is reduced in the caudate, putamen and globus pallidus at post-mortem (Warren *et al.*, 2007*b*) and *in vivo* (Fig. 2A) (Seppi *et al.*, 2006). Dopamine levels are reduced in the putamen, caudate nucleus, substantia nigra and globus pallidus at post-mortem (Fig. 2C) (Ruberg *et al.*, 1985; Hornykiewicz and Shannak, 1994). *In vivo* PET and single photon emission computed tomography (SPECT) studies indicate reduced levels of D2 receptors in the basal ganglia (Fig. 2D) (Brooks *et al.*, 1992; Arnold *et al.*, 2002; Oyanagi, 2002) while post-mortem studies show corresponding loss of D2 receptors in the putamen, caudate and substantia innominata (Ruberg *et al.*, 1985; Pierot *et al.*, 1988; Pascual *et al.*, 1992; Landwehrmeyer and Palacios, 1994). One study reported higher D2 receptor binding in the striatum compared with controls (Warren *et al.*, 2007*a*), which might represent receptor upregulation in response to loss of presynaptic dopaminergic neurons. In contrast D1 receptors appear relatively well preserved (Pierot *et al.*, 1988). There is also evidence that the mesocortical pathway is impaired in PSP, with degeneration of dopaminergic neurons in the ventral tegmental area (Murphy *et al.*, 2008) and loss of dopamine receptors in the frontal cortex, measured post-mortem with ^3^H-spiperone (Fig. 2B) (Ruberg *et al.*, 1985). This is especially relevant to the often profound change in motivation and apathy in PSP.

In contrast to Parkinson’s disease, motor symptoms in typical clinical presentations of PSP (increasingly known as progressive supranuclear palsy-Richardson’s syndrome, or PSP-RS, to distinguish it from other phenotypes of PSP pathology) (Höglinger *et al.*, 2017) typically do not respond well to dopaminergic therapy. This may be because in PSP there is loss of both dopaminergic neurons and receptors in the basal ganglia and cerebral cortex. This contrasts with Parkinson’s disease, in which predominant loss of presynaptic nigrostriatal dopaminergic neurons is greater than the relative preservation, or even upregulation, of post-synaptic dopamine receptor densities (Olanow, 2004).

### Corticobasal syndrome

CBS is caused by corticobasal degeneration (CBD) pathology in about 60% of cases, the remainder being due to PSP, FTD, Alzheimer’s disease and other pathology (Boeve *et al.*, 1999; Alexander *et al.*, 2014). Patients with CBD have pathological neuroglial tau deposits, severe neuronal loss and gliosis in the substantia nigra and striatum, typically with a history of extrapyramidal signs (Oyanagi *et al.*, 2001; Armstrong *et al.*, 2013; Coyle-Gilchrist *et al.*, 2016). Despite this, the *in vivo* imaging evidence of dopaminergic deficits is inconsistent. Fluorodopa PET indicates presynaptic dopaminergic reductions in the caudate, putamen and frontal cortex (Sawle *et al.*, 1991; Nagasawa *et al.*, 1996; Laureys *et al.*, 1999; Klaffke *et al.*, 2006; Pirker *et al.*, 2015), but with wide variation and surprisingly no correlation with disease duration or severity (Cilia *et al.*, 2011). Indeed some patients with autopsy-confirmed CBD have had a normal dopamine transporter SPECT scan despite prominent parkinsonian features (Chaal and Rowe, 2013; Kaasinen *et al.*, 2013), and D2 receptor levels can be unchanged (Klaffke *et al.*, 2006; Pirker *et al.*, 2013). These conflicting results may partly reflect the poor clinicopathological correlation of CBS with CBD (Boeve *et al.*, 1999; Cilia *et al.*, 2011; Alexander *et al.*, 2014). This is arguably a greater problem in the older literature, which often used CBD when referring to CBS, and therefore may include a high proportion of Alzheimer’s disease in their cases. We suggest that future studies of CBS need corollary pathological or biomarker evidence to distinguish CBD and non-CBD causes of CBS. The current evidence suggests a complex and inconsistent relationship between nigrostriatal dopamine deficiency and symptoms in patients with CBS, but evidence is scarce in comparison to other disorders.

## Noradrenaline

**Figure 3:**
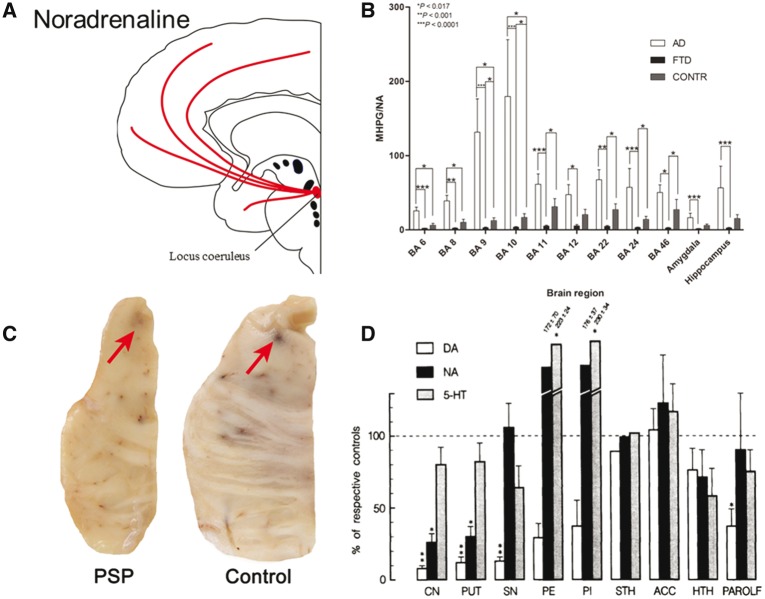
**Noradrenergic deficits in FTD and PSP.** (**A**) Schematic illustration of noradrenergic pathways. (**B**) MHPG/noradrenaline ratios, indicative of catabolic noradrenergic turnover, are reduced in Brodmann areas 11, 22, 24 and 46 in FTD. From Vermeiren *et al.* (2016). Reprinted with permission from the authors and IOS Press. The publication is available at IOS Press through http://dx.doi.org/10.3233/JAD-160320. (**C**) Post-mortem brainstem tissue from control and PSP brains. There is a paler locus coeruleus suggesting loss of melatonin-containing noradrenergic neurons. Courtesy of Kieran Allison, Cambridge Brain Bank. (**D**) Noradrenaline levels are reduced in the caudate (CN), putamen (PUT), hippocampus (HTH) and parolfactory cortex (PAROLF). Serotonin levels are reduced in those areas as well as in the subthalamic nucleus (SN). Dopamine levels are reduced in those areas as well as the globus pallidus externa (GPe) and interna (GPi). From Hornykiewicz and Shannak (1994). Reprinted with permission from Springer.

The locus coeruleus in the pons is the principle site of noradrenaline synthesis in the brain and contains the soma of noradrenergic neurons that project to the forebrain (Fig. 3A). Different subpopulations of neurons within the locus coeruleus project to the orbitofrontal, medial prefrontal, anterior cingulate and motor cortices (Chandler *et al.*, 2014). These noradrenergic pathways have an important role in regulating the function of the prefrontal cortex (McGaughy *et al.*, 2008; Chandler *et al.*, 2014), while in contrast to dopamine, there is minimal noradrenergic innervation of the striatum. Noradrenaline acts via α and β G protein coupled receptor families, each of which comprise subtypes that have different responses to ligand binding (Sara, 2009). The effect of noradrenaline depends on the relative densities of these receptors (Aston-Jones and Cohen, 2005). For example, noradrenergic input to the basal forebrain can promote arousal by activating cholinergic neurons through α1 and β1 receptors and inhibiting GABAergic neurons through α2 receptors (Schwarz and Luo, 2015), while presynaptic auto-inhibitory α2 receptors may paradoxically enhance noradrenergic transmission in response to antagonists (Invernizzi and Garattini, 2004). Noradrenaline is involved in regulating a range of behaviours including wakefulness, attention, memory and decision-making (Rowe *et al.*, 1996; Sara, 2009; Dalley *et al.*, 2011; Aston-Jones and Waterhouse, 2016). In comparative models, for example rats, limiting noradrenergic transmission results in impaired executive function (Newman *et al.*, 2008; Chandler *et al.*, 2014) and increasing noradrenaline levels reduces impulsivity (Robinson *et al.*, 2008). Computational and neurophysiological models suggest noradrenergic pathways mediate salience and shift in attention (Aston-Jones and Cohen, 2005).

### Frontotemporal dementia

There is limited evidence for noradrenergic changes in FTD but in many respects, the noradrenergic pathways appear to be normal or near normal, relative to the marked deficits seen in other neurotransmitter pathways. For example, neuropathological studies of FTD suggest the preservation of cell density in the locus coeruleus, and noradrenaline levels are normal or even elevated in the frontal lobe (Vermeiren *et al.*, 2016), despite the presence of pathological tau inclusions (Nagaoka *et al.*, 1995; Yang and Schmitt, 2001; Brunnström *et al.*, 2011; Irwin *et al.*, 2016). However, there may be reduced noradrenaline catabolism and turnover. For example, one study found low 3-methoxy-4-hydroxyphenylglycol (MHPG) to noradrenaline ratios, a proposed marker of noradrenergic turnover, in the frontal and temporal lobes, anterior cingulate, amygdala and hippocampus (Fig. 3B) (Vermeiren *et al.*, 2016). In contrast, several studies show normal levels of noradrenaline and MHPG in CSF (Sjogren *et al.*, 1998; Engelborghs *et al.*, 2008; Vermeiren *et al.*, 2013). However, in one of these studies there was a correlation between CSF levels of noradrenaline and disease severity, even though overall levels were unchanged (Engelborghs *et al.*, 2008). The enzyme monoamine oxidase, which metabolizes noradrenaline, is reduced in some areas of the brain (including the temporal lobe) although levels are unchanged in the frontal lobe (Sparks *et al.*, 1991). This anatomical heterogeneity may be one reason for the inconsistent reports of MHPG/noradrenaline levels. However, an alternative explanation is that the locus coeruleus receives inhibitory serotoninergic innervation from the upper raphe nuclei (Yang and Schmitt, 2001) such that the major loss of serotonergic projections in FTD (see below) serves indirectly to increase noradrenaline signalling to the frontal lobe.

Idazoxan is an α2 adrenoceptor antagonist that increases synaptic noradrenaline levels by antagonism of inhibitory autoreceptors on noradrenergic neurons. Idazoxan improved attention, planning and problem-solving in a small group of patients with FTD (Sahakian *et al.*, 1994; Coull *et al.*, 1996). Looking ahead to candidate symptomatic therapies, selective noradrenergic reuptake inhibitors such as atomoxetine and reboxetine, or combined serotonin and noradrenaline reuptake inhibitors like venlafaxine and duloxetine, may provide better tolerated augmentation of noradrenergic neurotransmission in FTD building on the evidence of their safety and efficacy in other disorders (Wang *et al.*, 2011; Cubillo *et al.*, 2014; Kehagia *et al.*, 2014; Ye *et al.*, 2015; Rae *et al.*, 2016).

### Progressive supranuclear palsy and corticobasal syndrome

Evidence is emerging of an early noradrenergic deficit in PSP, with loss of noradrenergic neurons and low noradrenaline levels in the basal ganglia. There is significant pathology in the locus coeruleus with both tau deposition (Dickson, 1999; Arnold *et al.*, 2013), and neuronal loss (Fig. 3C) (Hauw *et al.*, 1994; Mori *et al.*, 2002; Dickson *et al.*, 2010). A single post-mortem study also found reduced levels of noradrenaline in the caudate and putamen (Hornykiewicz and Shannak, 1994) (Fig. 3D), although noradrenergic receptor density is normally low in the striatum compared to cortex. These early and sometimes severe noradrenergic changes may be directly linked to cognitive and behavioural manifestations of PSP, such as rigidity and impulsivity, analogous to the treatable noradrenergic deficit underlying aspects of impulsivity in Parkinson’s disease (Kehagia *et al.*, 2014; Rae *et al.*, 2016). In keeping with this, a double-blind cross-over study of the α2 antagonist idazoxan showed improvement in motor function in PSP (Ghika *et al.*, 1991). However a larger study with a more potent α2 antagonist (efaroxan) found no effect (Rascol *et al.*, 1998). Atomoxetine has been shown to reduce impulsivity and executive deficits in Parkinson’s disease (Marsh *et al.*, 2009; Kehagia *et al.*, 2014; Ye *et al.*, 2015), but evidence is lacking in PSP. Evidence is also lacking for noradrenergic changes in CBS, although tau pathology is present in the locus coeruleus (Dickson, 1999).

## Serotonin

**Figure 4:**
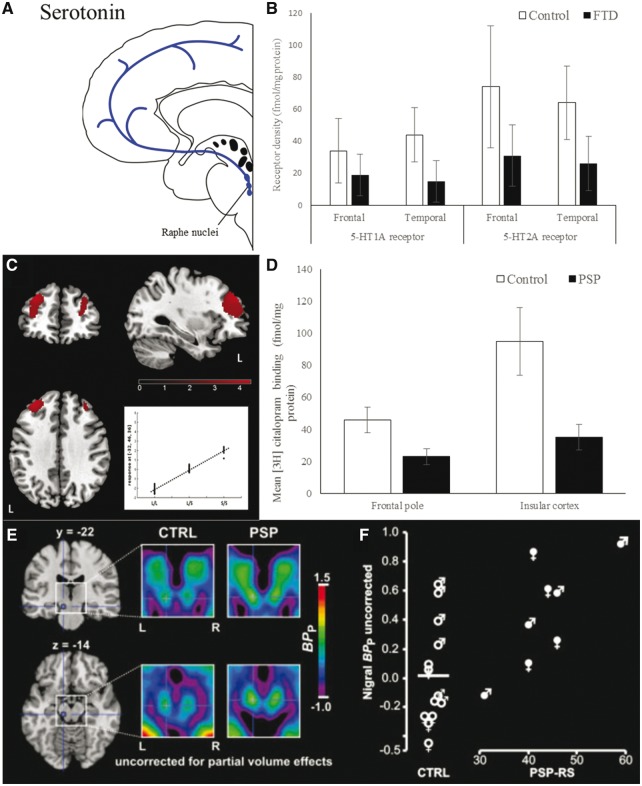
**Serotonergic deficits in FTD and PSP.** (**A**) Schematic illustration of serotonin pathways. (**B**) 5-HT1 and 2A receptor density is reduced in the frontal and temporal lobe in FTD. Graph of data from Bowen *et al.* (2008). Reprinted with permission of the authors and Springer. (**C**) Effect of 5-HTTLPR genotype on brain perfusion in FTD patients. Comparison of long (L/L) versus short (S/S) carriers at the same disease stage showing reduced perfusion of some areas of the frontal lobe in L/L carriers. From Premi *et al.* (2015). Reprinted with permission from Elsevier. (**D**) Presynaptic serotonergic neurons (measured by citalopram binding to post-mortem tissue) are reduced in the frontal and insular cortices in PSP. Graph of data from Chinaclia and Landwehrmeyer (1993). Reprinted with permission from Elsevier. (**E**) 5-HT2A receptor PET binding is increased bilaterally in the striatum and substantia nigra compared with controls. In the same study (**F**) disease severity positively correlated with 5-HT2A binding potential in the striatum. From Stamelou *et al.* (2009). Reprinted with permission from Wiley.

Serotonin (5-HT) is synthesized mainly by two groups of neurons in the raphe nuclei in the brainstem, which project widely (Fig. 4A) (Charnay and Léger, 2010). The rostral group, comprising 85% of serotonergic neuron cell bodies, project to the cerebral cortex, thalamus, hypothalamus and basal ganglia (Hornung, 2003). The caudal group project mainly to the brainstem and spinal cord (Hornung, 2003). With these widespread projections, serotonin regulates many higher brain functions related to cognitive control, learning, and affect (Harvey, 2003; Ciranna, 2006; Canli and Lesch, 2007; Artigas, 2013). There are seven different serotonin receptor families (5-HT_1–7_), which are neuromodulatory G protein coupled receptors except for the 5-HT_3_ receptor family, which includes ligand-gated ion channels (Barnes and Sharp, 1999; Southan *et al.*, 2016). To add to this complexity, genetic polymorphisms within a receptor subtype (Barnes and Sharp, 1999) and presynaptic transporter (Porcelli *et al.*, 2012), influence serotonergic function.

Serotonin receptors are among the most complex and varied of neurotransmitter receptors, and while there is clear evidence of serotonergic deficits in FTLD, studies to date mainly lack a detailed breakdown of receptor subtypes, or focus on 1A and 2A receptors. Serotonin has important roles in synaptic plasticity and as a neuromodulator of the direct effects of other neurotransmitters (Celada *et al.*, 2013). For example, serotonin inhibits dopamine release and modulates glutamate and GABA transmission (Ciranna, 2006). In the hippocampus serotonin receptors reduce glutamate and stimulate GABA from inhibitory interneurons, reducing long term potentiation (Ciranna, 2006). In the frontal cortex glutamate release is inhibited by serotonin whereas in the prefrontal cortex serotonin enhances glutamate transmission (Dawson *et al.*, 2001; Ciranna, 2006). This suggests that FTLD-induced serotonin deficiency could cause widespread cognitive, motor and affective symptoms, directly and through the disruption of its modulation of other systems.

### Frontotemporal dementia

Serotonin dysfunction is a significant contributor to the behavioural and cognitive symptoms seen in bvFTD (Huey *et al.*, 2006; Hughes *et al.*, 2015). Reductions in serotonin transmission or postsynaptic receptor densities are associated with several symptoms seen in FTD including aggression, impulsivity, increased appetite and depression (Huey *et al.*, 2006). At post-mortem examination, 5HT1A and 2A receptors are reduced in the frontal and temporal lobes and the hypothalamus (Fig. 4B) (Sparks and Markesbery, 1991; Francis *et al.*, 1993; Procter *et al.*, 1999; Bowen *et al.*, 2008). *In vivo* PET studies corroborate the post-mortem findings with the 5-HT2A receptor binding potential reduced in the midbrain and medial frontal cortex (Franceschi *et al.*, 2005) and the 5-HT1A binding potential reduced across all cortical areas (Lanctôt *et al.*, 2007).

Evidence for actual serotonergic neuronal cell loss is less conclusive. One post-mortem study found loss of neurons in the raphe nucleus and their projections to the cerebral cortex, which correlated with disease duration (Yang and Schmitt, 2001). There is also pathological tau deposition in the raphe nuclei (Irwin *et al.*, 2016). This contrasts with other studies that report no change in imipramine binding, proposed as a measure of presynaptic serotonergic terminals (Sparks and Markesbery, 1991), while post-mortem biochemical assays of serotonin are normal or elevated in FTD (Bowen *et al.*, 2008; Vermeiren *et al.*, 2016) and CSF measures of serotonin and its metabolites are unchanged (Engelborghs *et al.*, 2008). Nonetheless, CSF homovanillic acid/5-hydroxyindoleacetic acid (HVA/5-HIAA) levels (a proposed marker of the serotonergic modulation of dopaminergic neurotransmission) correlate with aggressive behaviour in FTD (Engelborghs *et al.*, 2004, 2008). 5-HIAA/5-HT ratios (a proposed marker of serotonergic turnover) are also lower in FTD compared to controls in the frontal and temporal lobes (Vermeiren *et al.*, 2016). It is possible that these apparent inconsistencies between biochemical assays and receptor or neuronal markers result from different stages of serotonergic cell loss and downstream functional compensation. To test this hypothesis would require the comparison of methods within the same pathological cohort, preferably one that includes patients with a wide range of neurocognitive severity.

There appears to be an association between FTD and length polymorphism in the gene promotor S(5-HTTLPR) of the serotonin transporter gene (SLC6A4) which suggests serotonin may be involved in the pathogenesis of FTD. A short allele (5-HTTLPR-s) was associated with a greater susceptibility to FTD in one study (Albani *et al.*, 2008) although this was not replicated (Yokoyama *et al.*, 2015). The 5-HTTLPR variant also affects brain atrophy in FTD. Patients with a long 5-HTTLPR allele have correspondingly greater atrophy and lower perfusion at equivalent disease stages (Fig. 4C) (Premi *et al.*, 2015) while the short allele is associated with more atrophy in the left inferior frontal gyrus and less in the right temporal lobe (Yokoyama *et al.*, 2015). The long allele may have a protective effect on cognitive presentation but is not associated with better prognosis (Borroni *et al.*, 2010).

In bvFTD there are reduced neurophysiological markers of inhibitory control and prefrontal cortical function, which are restored with the selective serotonin reuptake inhibitor citalopram in a placebo-controlled double-blind assessment (Hughes *et al.*, 2015). Several open label studies without placebo-control have shown improvement in behavioural symptoms with serotonergic drugs. For example, citalopram reduced disinhibition, irritability and depression (Herrmann *et al.*, 2012) and improved Frontal Assessment Battery test scores (Herrmann *et al.*, 2012) and inappropriate sexual behaviour (Anneser *et al.*, 2007). Paroxetine improved behavioural symptoms in an open label study (Moretti *et al.*, 2003*a*) but this was not supported by a subsequent placebo-controlled blinded study (Deakin *et al.*, 2004). Trazodone may improve behavioural symptoms in bvFTD based on a randomized control cross-over study (Lebert *et al.*, 2004). Interestingly, trazodone differs from selective serotonin reuptake inhibitors (SSRIs): it is an antagonist of a range of serotonin receptors apart from 5HT1A where it is an agonist, and it inhibits the serotonin transporter. A meta-analysis of antidepressants in FTD showed a combined mean reduction of 15 points on the Neuropsychiatric Inventory, noting, however, that the evidence was mainly from small, non-placebo controlled trials (Huey *et al.*, 2006).

### Progressive supranuclear palsy and corticobasal syndrome

Pathological tau inclusions are found post-mortem in the raphe nuclei with PSP (Revesz *et al.*, 1996) while presynaptic serotonergic neurons are reduced in the caudate nucleus, frontal and temporal cortex (Fig. 4D) (Chinaclia and Landwehrmeyer, 1993). Serotonin levels were not significantly reduced in one post-mortem study (Hornykiewicz and Shannak, 1994). PET and post-mortem studies have both shown upregulation of 5-HT1B and 2A receptors in the substantia nigra and striatum (Fig. 4E) (Castro *et al.*, 1998; Stamelou *et al.*, 2009), which might represent compensation for loss of presynaptic serotonergic neurons. This upregulation correlated with severity of motor impairment (Fig. 4F) (Stamelou *et al.*, 2009), but information on the correlation with cognitive, affective or associative functions is also needed.

There have been case reports of patients with PSP showing some improvements in motor function with an SSRI (Miyaoka *et al.*, 2002), and anecdotal reports of serotonergic reuptake inhibition as an effective treatment for emotional lability (Rittman *et al.*, 2016). Overall there is not strong evidence for the efficacy of serotonergic drugs in PSP (Stamelou and Höglinger, 2016). This lack of evidence may be because studies have focussed on depression and anxiety as outcomes of treatment, rather than impulsivity, disinhibition or cognitive change (Rittman *et al.*, 2016).

There is neuronal loss and gliosis in the raphe nucleus in CBD (Gibb *et al.*, 1989). However *in vivo* data are lacking on the serotonergic pathways and receptor density in CBS, and there are no systematic trials of serotonin reuptake inhibitors.

## Acetylcholine

**Figure 5:**
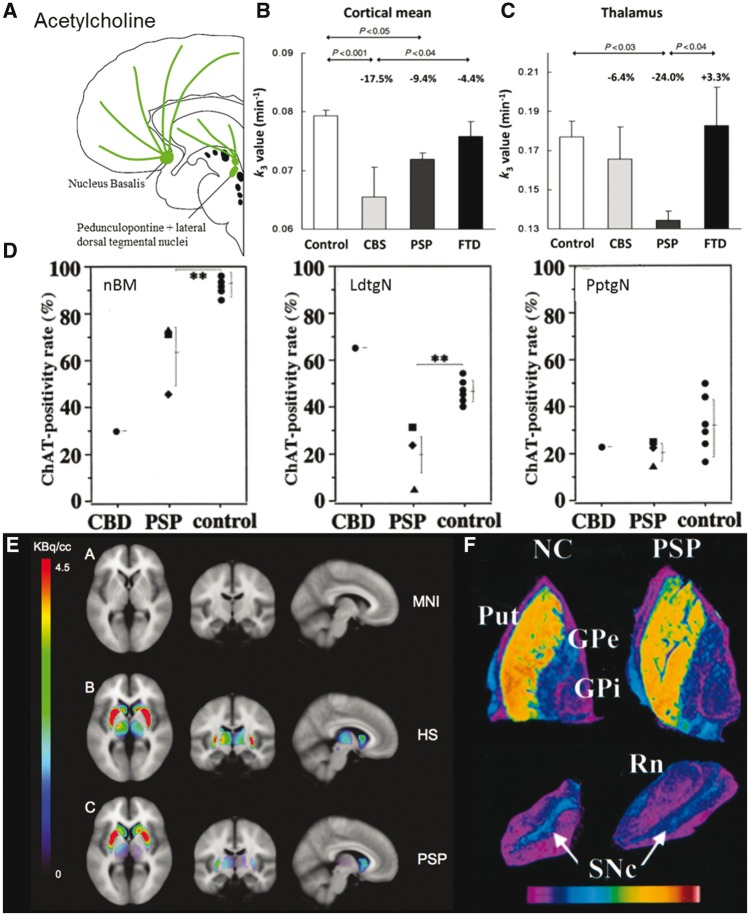
**Cholinergic deficits in FTD, PSP and CBS.** (**A**) Schematic illustration of cholinergic pathways. (**B** and **C**) ^11^C-MP4A PET, a measure of acetylcholinesterase activity, in healthy controls, CBS, PSP and FTD. Cortical k_3_ (a measure of PET ligand binding) is reduced in CBS and PSP but not FTD. Thalamic mean k_3_ is reduced in PSP but not CBS or FTD. From Hirano *et al.* (2010). Reprinted with permission from Oxford University Press. (**D**) Quantitative estimation of choline acetyltransferase (ChAT) positivity rate (%) in the nucleus basalis of Meynert (nBM), laterodorsal tegmental (LdtgN) and pedunculopontine tegmental (PptgN) nuclei. From Kasashima and Oda (2003). Reprinted with permission of Springer. (**E**) SPECT of acetylcholine transporter. MNI = MRI template; HS = healthy subject. Specific binding in the striatum, thalamus and pedunculopontine nucleus extracted by subtracting reference from region of interest binding. Binding is lower in the thalamus and pedunculopontine nucleus. From Mazere *et al.* (2012). Reproduced with permission from the Radiological Society of North America. (**F**) Autoradiogram of brain tissue from a healthy control (NC) and PSP. ^3^H-vesamicol binding to acetylcholine transporter (VAChT). There is reduction in binding in the putamen (Put) and substantia nigra pars compacta (SNc). Rn = red nucleus. Image intensity converted to pseudocolour representation according to key. From Suzuki *et al.* (2002). Reproduced with permission from Wolters Kluwer.

Acetylcholine is neuromodulatory on many areas of the forebrain (Everitt and Robbins, 1997), and influences a wide range of cognitive functions including attention, memory and emotion, but also motor control, through cortical and subcortical transmission in the cortico-striato-thalamocortical circuits (Picciotto *et al.*, 2012). The major cholinergic inputs to the cerebral cortex originate in the nucleus basalis of Meynert and adjacent nuclei in the basal forebrain (Fig. 5A) (Selden, 1998). Two other cholinergic nuclei in the brainstem, the pedunculopontine and lateral dorsal tegmental nuclei, project to the thalamus. Acetylcholine acts on two main receptor classes in the brain; muscarinic G protein coupled receptors (M1–5) and nicotinic ligand-gated ion channels (Picciotto *et al.*, 2012). Cholinergic receptors can have excitatory or inhibitory effects depending on their subtype and pre- versus postsynaptic location (Picciotto *et al.*, 2012).

Cholinergic drugs are in widespread use clinically, although not specifically in FTLD. For example, anti-cholinergic drugs reduce tremor and dystonia in movement disorders (Rifkin *et al.*, 1978), although they can cause impairments in learning and memory (Everitt and Robbins, 1997). The loss of cholinergic neurons and reduced choline acetyltransferase in Alzheimer’s disease (Francis *et al.*, 1999) lies behind the widespread use of cholinesterase inhibitors to enhance cholinergic transmission and thereby alleviate cognitive symptoms in Alzheimer’s disease (Rogers *et al.*, 1998). This cholinergic hypothesis has led to research into the role of cholinergic therapies in other dementias, including syndromes arising from FTLD.

### Frontotemporal dementia

Cholinergic pathways are affected in FTD but not to the same extent as in Alzheimer’s disease. While there is some loss of cholinergic neuronal markers in the nucleus basalis, overall cholinergic pathways to the cortex appear unaffected. Choline acetyltransferase, the enzyme for the synthesis of acetylcholine, can be used as a marker of presynaptic cholinergic neuron integrity. Post-mortem levels of choline acetyltransferase are reduced in the nucleus basalis of Meynert and the hypothalamus but are normal in the frontal, temporal and parietal lobes (Wood *et al.*, 1983; Hansen *et al.*, 1988; Sparks and Markesbery, 1991; Procter *et al.*, 1999). Acetylcholinesterase, which catalyses the breakdown of acetylcholine, is predominantly located on the presynaptic cholinergic neurons. Levels are reduced in the nucleus basalis at post-mortem (Sparks and Markesbery, 1991) but have been normal in the thalamus and cerebral cortex when measured *in vivo* with ^11^C-MP4A PET (Fig. 5B and C) (Hirano *et al.*, 2010) or at post-mortem (Meier-Ruge *et al.*, 1984; Sparks and Markesbery, 1991).

Studies are inconsistent on cholinergic receptors in bvFTD. ^123^IQNB SPECT imaging of two patients with Pick’s disease indicated reduced muscarinic receptor density in the frontal and temporal cortex (Weinberger *et al.*, 1991) consistent with autoradiography in a case report (Yates *et al.*, 1980). In contrast two studies found no significant change in muscarinic receptor density post-mortem (Wood *et al.*, 1983; Procter *et al.*, 1999).

There is evidence of a cholinergic deficit in primary progressive aphasia. In patients with semantic dementia there was loss of muscarinic receptors in the temporal lobe (Odawara *et al.*, 2003). Disproportionate atrophy of the basal forebrain nuclei was identified in a high resolution MRI study, most evidently in the semantic variant, and to a lesser extent the non-fluent variant (Teipel *et al.*, 2016). This is relevant in view of the evidence that the frontotemporal language networks of a healthy brain receive significant cholinergic inputs (Amunts *et al.*, 2010). The logopenic variant had minimal structural change, despite its strong clinicopathological correlation with Alzheimer’s disease.

Despite the possible cholinergic deficits in bvFTD and PPA, cholinesterase inhibitors do not convincingly improve cognitive function. An open label non-randomized study found that behavioural changes improved with rivastigmine, in comparison to a group that took antipsychotics and benzodiazepines (Moretti *et al.*, 2004). In contrast bvFTD patients taking donepezil had worsening disinhibition and compulsive behaviour (Mendez *et al.*, 2007). A randomized, double-blind trial of galantamine versus placebo found no effect on cognitive function or activities of daily living (Kertesz *et al.*, 2008).

### Progressive supranuclear palsy and corticobasal syndrome

There are marked cholinergic deficits in PSP, which may contribute not only to cognitive impairment but also postural instability via the pedunculopontine nucleus (Jellinger, 1988; Warren *et al.*, 2005). There is loss of cholinergic neurons and their presynaptic terminals in many subcortical regions in PSP. Choline acetyltransferase is reduced in the nucleus basalis of Meynert, midbrain nuclei and pedunculopontine nucleus (Fig. 5D) (Juncos *et al.*, 1991; Javoy-Agid, 1994; Kasashima and Oda, 2003) as well as the putamen, caudate and pallidum (Ruberg *et al.*, 1985; Pierot *et al.*, 1988; Javoy-Agid, 1994). Presynaptic acetylcholine transporters are reduced in the putamen and substantia nigra, while sparing the globus pallidus and cerebral cortex (Fig. 5F) (Suzuki *et al.*, 2002). ^123^I-IBVM SPECT, which binds to acetylcholine transporters, reveals reduced signal in the thalamus of PSP patients (Fig. 5E) (Mazere *et al.*, 2012) and PET studies show reduced acetylcholinesterase binding in the pons, basal ganglia and thalamus (Shinotoh *et al.*, 1999; Gilman *et al.*, 2010; Hirano *et al.*, 2010).

There is loss of cholinergic projections from the brainstem (pedunculopontine and laterodorsal tegmental nuclei) to the thalamus (Hirsch *et al.*, 1987; Jellinger, 1988; Kasashima and Oda, 2003). The pedunculopontine loss is especially relevant to the impairment of movement, gait and muscle tone in PSP (Benarroch, 2013). Deep brain stimulation of the pedunculopontine nucleus has been reported to improve PSP motor symptoms in selected cases, but definitive trials are lacking (Hazrati *et al.*, 2012; Servello *et al.*, 2014). One study reported that acetylcholine receptors are relatively well preserved in the striatum (Ruberg *et al.*, 1985) while other studies report a reduction in muscarinic and nicotinic receptors in the striatum (Landwehrmeyer and Palacios, 1994; Warren *et al.*, 2007*b*). With such small series, and variable methods, it is unclear if technical or phenotypic differences account for these inconsistencies.

There is also some limited evidence for cholinergic deficits in the cerebral cortex. Acetyltransferase levels are reduced in frontal cortex of PSP patients compared with controls both at post-mortem and with *in vivo* PET imaging (Ruberg *et al.*, 1985; Javoy-Agid, 1994; Hirano *et al.*, 2010). However, cortical muscarinic receptor levels appear to be unaffected in PSP, with levels similar to controls in PET studies (Ruberg *et al.*, 1985; Asahina *et al.*, 1998).

In clinical practice, cholinergic blockade with hyoscine is sometimes used for sialorrhoea and drooling, but it may worsen gait and memory in PSP (Litvan *et al.*, 1994). Despite this deleterious effect of anti-cholinergic medication, the converse ‘pro-cholinergic’ treatment by cholinesterase inhibitors is typically ineffective (Stamelou and Höglinger, 2016). A case series of rivastigmine in five patients found that it improved working memory, memory and verbal fluency but worsened motor function (Liepelt *et al.*, 2010). A randomized, placebo-controlled crossover study of donepezil showed no effect on quality of life, Progressive Supranuclear Palsy Rating Scale or global cognitive function (Litvan *et al.*, 2001). This study did find a slight improvement in one memory task but also worsened motor activities of daily living (Litvan *et al.*, 2001). Interestingly, the syndrome of pure akinesia and gait freezing, now recognized as a prodromal variant of PSP (Höglinger *et al.*, 2017) has been reported to improve after cholinesterase inhibition in an open case series (Kondo, 2006). Despite this encouraging study, replication in a placebo controlled trial is awaited.

In a post-mortem study of a single case of CBD the number of cholinergic acetyltransferase positive neurons in the nucleus basalis of Meynert was reduced (Kasashima and Oda, 2003). This was replicated *in vivo*, with reduced acetylcholinesterase levels in the frontal, parietal and occipital cortex (Hirano *et al.*, 2010). There is insufficient data on cholinergic treatment of patients with CBS, although it should be noted that ∼20–40% of patients with CBS have Alzheimer’s-type pathology not CBD (Boeve *et al.*, 1999; Alexander *et al.*, 2014). It is plausible, but not proven, that the Alzheimer pathology cases of CBS would respond better to cholinesterase inhibitors despite appearing similar to CBD cases in other clinical features. We therefore anticipate that clinical trials of CBS will stratify treatment according to biomarkers, such as amyloid PET imaging or CSF, to distinguish CBD from Alzheimer’s disease aetiology.

## Glutamate

Glutamate is the principle excitatory neurotransmitter in the brain. Glutamate acts on fast, short acting ionotropic receptors and slower but longer acting metabotropic glutamate receptors (mGluR) (Meldrum, 2000). The three main ionotropic glutamate receptors are named after the selective agonists *N*-methyl d-aspartate (NMDA), α-amino-3-hydroxyl-5-methyl-isoxazolepropionic acid (AMPA) and kainite (Meldrum, 2000). Glutamate has an important role in learning and memory formation. For example, NMDA receptors in the hippocampus regulate long term potentiation (Morris *et al.*, 1986; Rowland *et al.*, 2005) while sustained activation of the dorsolateral prefrontal cortex during working memory requires NMDA stimulation (Wang *et al.*, 2013). NMDA receptor antagonists impair attention, reaction time, processing speed and working memory in healthy humans (Malhotra *et al.*, 1996; Newcomer *et al.*, 2000), and may exacerbate psychotic symptoms (Gilmour *et al.*, 2012). Glutamate signalling through NMDA receptors is required to create and maintain gamma oscillations (Carlé *et al.*, 2011), which support many higher cognitive functions (Lange *et al.*, 1997; Bartos *et al.*, 2007; Williams and Boksa, 2010; Gaetz *et al.*, 2012; Gorelova *et al.*, 2012).

While glutamatergic transmission is essential for cognition, excessive glutamatergic transmission may also be harmful, promoting excitotoxic neuronal death (Mark *et al.*, 2001) that contributes to neurodegeneration in models of Alzheimer’s disease (Danysz *et al.*, 2000; Kalia *et al.*, 2008). It is possible that FTLD is similarly affected. Functionally, continuous overactivation of NMDA receptors alters the efficacy of information processing by reducing the sensitivity of neural networks and impairing their ability to detect a relevant signal from upstream neurons (Danysz *et al.*, 2000). Memantine is a low affinity NMDA receptor antagonist and selectively blocks pathological tonic NMDA receptor activation (associated with amyloid plaques) without preventing NMDA-mediated synaptic transmission. In addition to potential symptomatic effects on cognition (Reisberg *et al.*, 2003), it might therefore also reduce chronic glutamatergic excitotoxicity (Danysz and Parsons, 2012).

### Frontotemporal dementia

There is preclinical and clinical evidence that glutamate is important in the pathogenesis of FTD. For example, transgenic mice that express pathological human tau have repetitive and disinhibited behaviour, coupled with NMDA receptor hypofunction (Warmus *et al.*, 2014). Treatment with an NMDA agonist restores their behaviour. Transgenic mice expressing mutations in the FTD-associated gene *CHMP2B*, have altered AMPA receptor composition (Gascon *et al.*, 2014), with impaired sociability, which can be reversed if normal AMPA receptor composition is restored (Gascon *et al.*, 2014). Mouse models expressing pathological human tau suggest glutamate mediated excitotoxicity could accelerate neuronal loss in tauopathies such as FTD (Decker *et al.*, 2016). These preclinical studies raise the possibility that pharmacological glutamatergic treatments might reduce symptom severity and improve prognosis.

**Figure 6:**
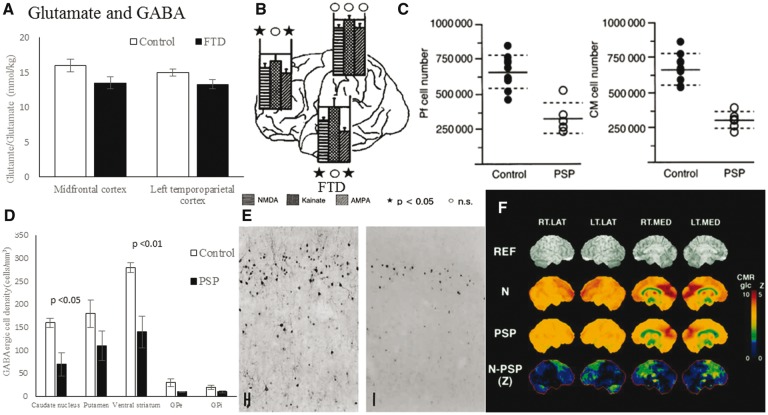
**Glutamate and GABA deficits in FTD and PSP.** (**A**) Mean metabolite concentrations using magnetic resonance spectroscopy. Glutamine–glutamate concentrations are reduced in the frontal cortex of FTD. Graph of data from Ernst *et al.* (1997). Reprinted with permission from the authors and the Radiological Society of North America. (**B**) Post-mortem glutamatergic receptor binding in FTD. Binding to NMDA and AMPA receptors is reduced in the frontal and temporal lobes. From Procter *et al.* (1999). Reprinted with permission from S. Karger AG. (**C**) Neuron number in two thalamic nuclei [parafascicular (Pf) and centromedian (CM)] that contain glutamatergic neurons is reduced in PSP compared with controls. Adapted from Henderson *et al.* (2000), with permission from the authors and Oxford University Press. (**D**) Numbers of GABAergic neurons (measured by glutamic acid decarboxylase mRNA expression) in the striatum and pallidum in controls and PSP patients. There is significant reduction in striatal GABAergic neurons in patients. Graph of data from Levy *et al.* (1995), reprinted with permission from the authors and Wolters Kluwer. (**E**) Calbindin immunohistochemistry of GABAergic cells in the frontal cortex of FTD and control brains. From Ferrer (1999). Reproduced with permission from Karger. (**F**) ^11^C-flumazenil PET binding to benzodiazepine receptors in healthy controls (N), PSP and the group difference in cortical and subcortical areas. From Foster *et al.* (2000). Reproduced with permission from Wolters Kluwer.

In patients, glutamatergic pyramidal neurons are reduced in the thalamus, frontal and temporal cortex (Ferrer, 1999). Magnetic resonance spectroscopy of patients with FTD has found glutamate/glutamine levels are reduced in the frontal and temporal lobes (Fig. 6A) (Ernst *et al.*, 1997; Sarac *et al.*, 2008). There is an inverse correlation between CSF glutamate levels and verbal agitation (Vermeiren *et al.*, 2013).

Both ionotropic and metabotropic glutamate receptors are affected in FTD. For example, AMPA and NMDA receptor densities are reduced in the frontal and temporal lobes of patients at post-mortem (Francis *et al.*, 1993; Procter *et al.*, 1999; Bowen *et al.*, 2008), while AMPA receptor composition is also abnormal (Fig. 6B) (Gascon *et al.*, 2014). Using the ligand ^11^C-ABP688, PET of patients with bvFTD found reduced availability of metabotropic glutamate receptors (mGluR5) in the frontal and temporal lobes, basal ganglia and thalamus (Leuzy *et al.*, 2016). However, one study found that post-mortem levels of metabotropic glutamate receptors type 1 and 5 (mGluR1 and 5) are increased in the frontal cortex (Dalfo *et al.*, 2005).

A phase II randomized placebo-controlled trial of memantine showed no benefit in patients with bvFTD (Boxer *et al.*, 2013). A double-blind placebo-controlled crossover trial of memantine in PPA was also negative (Johnson *et al.*, 2010). However, these studies were not powered to detect small treatment effects. While there may be no true benefit, it remains possible that small treatment effects exist which would be amplified if other neurotransmitter deficits were also normalized, in particular GABAergic impairments. The GABA–glutamate interaction is of particular relevance because it supports precisely tuned oscillatory dynamics of neural circuits for cognition (Bastos *et al.*, 2012).

### Progressive supranuclear palsy and corticobasal syndrome

Loss of glutamatergic neurons in the basal ganglia may partly explain why dopaminergic therapy is ineffective in PSP. Glutamate modulates dopamine release and loss of glutamatergic neurons may prevent patients compensating for dopaminergic neuron loss (Lange *et al.*, 1997). Glutamatergic neurons from the caudal intralaminar nuclei that form the thalamostriatal pathway are reduced in PSP (Fig. 6C) (Henderson *et al.*, 2000). However, the severity of this neuronal loss does not correlate with disease duration or severity (Henderson *et al.*, 2000). In contrast, NMDA receptor levels are preserved in the frontal and temporal lobes and striatum (Holemans *et al.*, 1991).

Glutamatergic over-activity is implicated in Parkinson’s disease and by analogy has been considered a candidate mechanism of accelerated neurodegeneration in PSP (Lange *et al.*, 1997). Amantadine is an NMDA receptor antagonist that is often used to treat motor symptoms (Kompoliti *et al.*, 1998; Stamelou and Höglinger, 2016), although there is no randomized controlled trial evidence of efficacy in PSP. Gabapentin has complex pharmacodynamics and in part acts by increasing GABA and reducing glutamate levels (Sills, 2006). A randomized blinded trial of gabapentin in 14 patients found no effect on motor function but improved outcome in anti-saccade control (Poujois *et al.*, 2007), which is associated with frontal lobe integrity (Mirsky *et al.*, 2011; Perneczky *et al.*, 2011) and commonly impaired in PSP (Garbutt *et al.*, 2008; Zhang *et al.*, 2016). There are no reports of post-mortem or *in vivo* glutamate measurements in CBS.

## Gamma-aminobutyric acid

GABA is the predominant inhibitory neurotransmitter in the brain, formed by glutamate decarboxylase conversion of glutamate to GABA in interneurons. There are two classes of GABA receptors: GABA_A_ ligand-gated ion channels and GABA_B_ G protein coupled neuromodulatory receptors. GABAergic inhibitory neurons dampen and balance excitation within neural circuits, but do more than simply counteract excitatory glutamatergic neurons. They have a key role in the regulation of oscillatory dynamics, including the generation of gamma oscillations and regulation of the magnitude and frequency of these oscillations (Owens and Kriegstein, 2002; Mann and Paulsen, 2007; Buzsáki and Wang, 2012). This is essential for coordinating information transfer and information processing in the brain (Fries, 2009; Bastos *et al.*, 2012). Increasing synaptic GABA levels increases gamma power during cognitive control tasks (Frankle *et al.*, 2009) whereas inhibiting GABA receptors reduces gamma oscillatory power and impairs inhibition and working memory (Hines *et al.*, 2013). Gamma oscillations correlate with GABA concentrations (as measured by magnetic resonance spectroscopy) in the visual (Muthukumaraswamy *et al.*, 2009), primary motor (Gaetz *et al.*, 2011) and dorsolateral prefrontal cortex (Kujala *et al.*, 2015) while GABA_A_ receptor density (as measured by flumazenil-PET) correlates with gamma frequency and magnitude (Kujala *et al.*, 2015). Impaired GABA neurotransmission has been implicated in a number of brain disorders including schizophrenia (Gonzalez-Burgos *et al.*, 2011) and Huntington’s disease (Reynolds and Sally, 1990) as well as the syndromes associated with FTLD.

### Frontotemporal dementia

The subgroup of GABAergic neurons that bind calbindin-D28k are reduced in upper neocortical layers of the frontal and temporal cortex in FTD (Ferrer, 1999), especially in layers II and III (Fig. 6E) (Ferrer, 1999). However, in the same study, the subgroup of GABAergic basket and chandelier neurons that bind parvalbumin were preserved (Ferrer, 1999). The superficial layers II and III are the main source of cortico-cortical feedforward efferent projections and receive feedback projections from deep layers. Gamma oscillations and coherence are reduced between the frontal lobes of patients with bvFTD (Hughes *et al.*, 2013), which may relate to loss of cortical feedforward information processing and cognitive decline (Mann and Paulsen, 2007). GABA concentrations are also decreased in the basal ganglia in bvFTD (Kanazawa *et al.*, 1988). GABAergic approaches to treatment of FTD symptoms warrant further investigation, but evidence of their clinical efficacy is currently lacking.

### Progressive supranuclear palsy and corticobasal syndrome

GABAergic interneurons are reduced in PSP. A post-mortem study found a 50–60% decrease in the number of GABAergic neurons (estimated from the number expressing glutamic acid decarboxylase mRNA, by *in situ* hybridization) in the caudate nucleus, putamen, ventral striatum and pallidum (Fig. 6D) (Levy *et al.*, 1995). Binding to GABA_A_ receptors is reduced in the globus pallidus but preserved in the striatum (Landwehrmeyer and Palacios, 1994; Suzuki *et al.*, 2002). A flumazenil-PET study showed loss of GABA_A_ receptors compared with controls (Fig. 6F) (Foster *et al.*, 2000).

There are case reports of GABA receptor agonists improving speech, eye movements, akinesia and rigidity in PSP (Daniele *et al.*, 1999; Cotter *et al.*, 2010; Dash, 2013; Chang and Weirich, 2014), but in the authors’ experience this phenomenon is very uncommon and there are no randomized placebo controlled studies. There are no reports of post-mortem or *in vivo* assessments of GABA in CBS.

## Towards better symptomatic treatment in frontotemporal lobar degeneration

Despite their overlapping clinical phenotypes and pathological features, the major clinical syndromes associated with FTLD have different neurotransmitter deficits (summarized in Table 1). Restoring these deficits, individually or in combination, has the potential to improve cognitive, behavioural and motor symptoms. However, the evidence base for therapeutic effects is dominated by small, open-label studies in unstratified populations.

To summarize the evidence for selective deficits, FTD causes loss of serotonergic and dopaminergic neurons and receptor densities, whilst noradrenergic and cholinergic pathways are relatively preserved. There is loss of both glutamatergic and GABAergic neurons but the functional consequence of their deficits is unclear, in part because of the complex and dynamic interaction between GABAergic and glutamatergic neurons in cortical circuits. In PSP, the most evident neurotransmitter deficits are dopaminergic, noradrenergic and cholinergic, whilst serotonergic projections appear to be relatively preserved. There is evidence of a glutamatergic and GABAergic deficit, which provide potential avenues for non-dopaminergic therapy. There is limited evidence on the neurotransmitter deficits in CBS, with some evidence of deficits in both cholinergic and dopaminergic pathways.

Although clinical trials and cases series have not shown consistent benefits from the modulation of neurotransmitters in FTLD syndromes, this may be due to weaknesses in research methodology rather than a true lack of effect. For example, many studies use what would now be considered as outdated and inaccurate diagnostic criteria, which reduces the applicability to contemporary patient populations. Many clinical studies are open-labelled and in small series, sometimes fewer than 10 patients, giving little power to detect benefits, let alone guide therapeutic stratification. There is a paucity of replication studies, and where studies contain a ‘conceptual replication’, details in research methodology confound the interpretation of seemingly conflicting results. Much of the research comes from post-mortem brain tissue, which has the advantage of providing concurrent pathological validation of the disorder. However, post-mortem studies have tended to use small series (*n* < 10), and by the nature of post-mortem material, they cannot provide insights into the early or sequential changes in neurotransmitter systems. Future work will benefit from longitudinal and *in vivo* studies, exploiting advances in PET ligands (Finnema *et al.*, 2015), ultra-high field MRI and spectroscopy (Agarwal and Renshaw, 2012), and CSF biomarkers. Early PET studies of necessity used non-specific ligands, which may not correspond to the receptor specificities of psychopharmacological agents. This is not to criticise either body of work, but it does impair the direct comparison of imaging and pharmacological studies, even where comparable patient groups are studied. Similarly, future preclinical studies would benefit from within-sample comparisons of different methods, seeking not only cross-validation of biochemical or receptor assays, but also the relationship between different measures, for example neuronal loss, receptor density, and biochemical turnover of a neurotransmitter. Such cross-modal studies would provide a powerful resource to model disease progression and functionally relevant compensatory changes in FTLD.

Further research is required into the effect of FTLD on different neurotransmitter receptors and their subtypes, not only to guide candidate drug selection, but also to determine the progression of changes from early to late stage disease. Without this detailed knowledge, there is a risk that a given drug may be effective at one stage of disease but be counterproductive at another. Such non-linear dose-response effects are common in dopaminergic treatments of Parkinson’s disease (Cools, 2006; Rowe *et al.*, 2008), but the principal of ‘U-shaped’ responses to drug treatment also affect serotonergic (Macoveanu *et al.*, 2013; Hughes *et al.*, 2015) and noradrenergic drugs (Ye *et al.*, 2015). Where drug effects follow a ‘U-shaped’ response, the focal nature of FTLD presents a special challenge. Take the behavioural variant of FTD as an example. If prefrontal and temporal cortex are deficient in a given neurotransmitter (whether neuronal density, receptor density, or afferent projections), but motor, parietal and occipital cortex are not, then any systemic treatment based on restoring that neurotransmitter in frontal and temporal cortex will risk ‘overdosing’ the unaffected areas. This problem is well established in Parkinson’s disease, in the sometimes difficult balance between motor disability and impulse control disorders (Napier *et al.*, 2015). The application of focal treatments to restore biochemical function, such as dopaminergic stem cell transplants or gene therapy to induce dopamine synthesis in striatal cells, can overcome some of the adverse consequences of systemic drug treatment in Parkinson’s disease. However, such localized treatments seem even more challenging in a diffuse lobar cortical disorder. Similarly, the use of Designer Receptors Exclusively Activated by Designer Drugs (DREADDs) to restore or enhance focal and selective neurotransmitter systems is having a major impact in drug discovery and systems neuroscience (Roth, 2016), but seems far from direct clinical applications. For the time being, systemic drug delivery is likely to be the mainstay of clinical therapeutics.

We suggest three steps to improve the likelihood of new and effective pharmacological treatments. First, clarification of the links between individual neurotransmitters and specific clinical end-points. We suggest that identifying the neurotransmitter deficits that correlate with clinical severity is essential to guide treatment studies. This evidence may draw on *in vivo* imaging and CSF studies and post-mortem immunohistochemistry of cases that have been regularly phenotyped during disease progression. This would be a considerable undertaking, but possible if added to existing longitudinal studies (Rohrer *et al.*, 2015*b*; Woodside *et al.*, 2016).

Second, it is essential to implement stratification of patients in future trials, selecting participants for their relevant symptoms rather than the diagnosis alone. For example, in a trial to demonstrate a clinical effect of serotonergic treatment on impulsivity in bvFTD, based on experimental medicines evidence (Hughes *et al.*, 2015), participants should not merely have bvFTD by consensus criteria, but also have impulsivity; noting that disinhibition is one of six criteria whereas only three are required for the diagnosis. Including patients with bvFTD who are not disinhibited is likely to reduce the power of a symptomatic treatment trial. Moreover, it may be better to include all patients with disinhibition arising from syndromes associated with FTLD in which disinhibition is common but not a diagnostic criterion (including semantic variant PPA, CBS and PSP) (Lansdall *et al.*, 2017). This would increase the power and relevance of the trial to a wider patient group.

Third, future clinical trials need careful selection of relevant outcome tools, especially where drugs are repurposed for new end-points. For example, selective serotonin reuptake inhibitors are licenced for affective disorders but it would be wrong to use a depression rating scale in bvFTD or PSP where the expected effect is on impulsivity. Similarly, cholinesterase inhibitors are licenced for Alzheimer’s disease for their effect on cognition but cognitive function scales would be inappropriate if the intended effect in say PSP were on gait and balance.

For each of the disorders associated with FTLD, it is likely that experimental medicines studies with biomarker based surrogate end-points are needed before randomized placebo controlled clinical trials are started. The evidence presented in this review suggests that there are strong grounds to pursue such experimental medicine studies, drawing on the preclinical psychopharmacology models and patient data, to minimize the risks of clinical trials. There are many candidate end-points, to demonstrate human target engagement and efficacy in the CNS. These may be used singly or in combination, including functional imaging; magnetic resonance spectroscopy (Cai *et al.*, 2012; Muthukumaraswamy *et al.*, 2013); PET imaging of neurotransmitters receptors and occupancy; magneto-/electro-encephalographic physiological indices of oscillatory dynamics (Muthukumaraswamy, 2014), focal function (Hughes *et al.*, 2015) and network interactions (Moran *et al.*, 2011, 2013; Hughes *et al.*, 2013; Gilbert *et al.*, 2016); CSF biomarkers; and neurocognitive batteries (Kehagia *et al.*, 2014).

This review has focused on the symptomatic benefits of restoring neurotransmitters. However, some of these agents, like trazodone, have wider effects on pathogenesis and neuronal survival that may also lead to disease modification or slowing of disease progression (Halliday *et al.*, 2017). Even where the principal effect is symptomatic, this may improve survival, such as the impact of dopaminergic therapy in Parkinson’s disease after its introduction in the late 1960’s (Uitti *et al.*, 1993). Relief of apathy, disinhibition, falls, and dementia in syndromes associated with FTLD might therefore improve survival as well as interim quality of life.

Finally, we note that there has been recent concern regarding international pharma investment in disorders of the CNS (Fineberg *et al.*, 2013). However, we suggest that there is scope and grounds for optimism for progress towards effective symptomatic pharmacological therapies. Such treatments, based on restoring neurotransmitter deficits, would reduce the cost, social and health burden of FTLD.

## Funding

This review was funded by the Holt Fellowship (A.M.), the Wellcome Trust (JBR 103838), and the National Institute for Health Research Cambridge Biomedical Research Centre and Cambridge Brain Bank.

## Supplementary material

Supplementary material is available at *Brain* online.

## Supplementary Material

Supplementary Data

## Abbreviations

AMPA: α-amino-3-hydroxyl-5-methyl-isoxazolepropionic acid
bvFTD: behavioural variant frontotemporal dementia
CBD: corticobasal degeneration
CBS: corticobasal syndrome
FTD: frontotemporal dementia
FTLD: frontotemporal lobar degeneration
NMDA: *N*-methyl d-aspartate
PPA: primary progressive aphasia
PSP: progressive supranuclear palsy
SPECT: single photon emission computed tomography
