# Supplementary material for: Neurotransmitter deficits from frontotemporal lobar degeneration
Source: Brain. 2018 Jan 24;141(5):1263–85. doi: 10.1093/brain/awx327 (PMC5917782; doi:10.1093/brain/awx327)
Supplement: Supplementary Data [file awx327_brain-2017-01233-file009.pdf]

Supplementary Table 1 – Neurotransmitter deficits in Frontotemporal Lobar Degeneration Syndromes

**Neurotransmitter**	**Frontotemporal dementia**	**Reference**	**Progressive supranuclear palsy**	**Reference**	**Corticobasal syndrome**	**Reference**
**Dopamine**						
Nigrostriatal pathway	Reduced dopaminergic neurons	(Rinne *et al.*, 2002)<br>(Sedaghat *et al.*, 2007)<br>(Pal *et al.*, 2001)<br>(Gil-Navarro *et al.*, 2013)	Reduced dopaminergic neurons	(Hardman *et al.*, 1997)<br>(Oyanagi *et al.*, 2001)<br>(Warren *et al.*, 2007)<br>(Baron *et al.*, 1986)<br>(Im *et al.*, 2006)<br>(Oh *et al.*, 2012)	Neuronal loss in substantia nigra and striatum	(Oyanagi *et al.*, 2001)<br>(Pirker *et al.*, 2015)<br>(Klaffke *et al.*, 2006)<br>(Laureys *et al.*, 1999)<br>(Sawle *et al.*, 1991)<br>(Nagasawa *et al.*, 1996)
	Low dopamine levels	(Kanazawa *et al.*, 1988)<br>(Nagaoka *et al.*, 1995)	Low dopamine levels	(Ruberg *et al.*, 1985)<br>(Hornykiewicz and Shannak, 1994)	Dopaminergic neuron loss not seen in all patients	(Cilia *et al.*, 2011)<br>(Chaal and Rowe, 2013)<br>(Kaasinen *et al.*, 2013)
			Reduced D2 receptors in the striatum	(Brooks *et al.*, 1992)<br>(Arnold *et al.*, 2002)<br>(Oyanagi, 2002)<br>(Pascual *et al.*, 1992)	D2 receptor levels are unaffected	(Klaffke *et al.*, 2006)<br>(Pirker *et al.*, 2013)
			Preserved D1 receptors	(Pierot *et al.*, 1988)		
Mesocortical pathway	Reduced D2 dopamine receptors	(Frisoni *et al.*, 1994)	Reduced dopaminergic neurons	(Murphy *et al.*, 2008)	Reduced dopaminergic neurons	(Sawle *et al.*, 1991)
	CSF dopamine reduced in some but not all studies	(Sjogren *et al.*, 1998)<br>(Vermeiren *et al.*, 2013)	Loss of D2 receptors in the frontal cortex	(Ruberg *et al.*, 1985)		
**Noradrenaline**						
	Minimal neuronal loss in	(Yang and Schmitt,	Neuronal loss in the locus	(Hauw *et al.*, 1994)	Tau pathology in the	(Dickson, 1999)

	the locus coeruleus	2001)<br>(Brunnström *et al.*, 2011)	coeruleus	(Mori *et al.*, 2002)<br>(Dickson *et al.*, 2010)	locus coeruleus	
	Noradrenaline levels normal or high in the cerebral cortex	(Vermeiren *et al.*, 2016)	Reduced levels of noradrenaline in the caudate and putamen	(Hornykiewicz and Shannak, 1994)		
	CSF noradrenaline correlates with disease severity	(Engelborghs *et al.*, 2008)				
**Serotonin**						
	Loss of neurons and tau deposition in the raphe nucleus	(Yang and Schmitt, 2001)<br>(Irwin *et al.*, 2016)	Tau deposition in the raphe nucleus	(Revesz *et al.*, 1996)	Neuronal loss in the raphe nucleus	(Gibb *et al.*, 1989)
	Serotonin transporter gene variants affect brain atrophy and clinical presentation	(Albani *et al.*, 2008)<br>(Yokoyama *et al.*, 2015)<br>(Premi *et al.*, 2015)	Serotonergic neurons reduced in the caudate and frontal and temporal lobes	(Chinaclia and Landwehrmeyer, 1993)		
	Serotonin levels normal or elevated at *post mortem*	(Bowen *et al.*, 2008)<br>(Vermeiren *et al.*, 2016)	Serotonin levels not significantly reduced	(Hornykiewicz and Shannak, 1994)		
	5HT1A and 2A receptors reduced in the midbrain, frontal and temporal lobes	(Sparks and Markesbery, 1991)<br>(Francis *et al.*, 1993)<br>(Procter *et al.*, 1999)<br>(Bowen *et al.*, 2008)<br>(Franceschi *et al.*, 2005)<br>(Lanctôt *et al.*, 2007)	5HT1B and 2A are upregulated in the basal ganglia	(Castro *et al.*, 1998)<br>(Stamelou *et al.*, 2009)		
	CSF serotonin levels unchanged but HVA/5HIAA levels correlate with aggressive behaviour	(Vermeiren *et al.*, 2016)<br>(Engelborghs *et al.*, 2008)				

**Acetylcholine**						
	Reduced cholinergic neurons in the nucleus basalis	(Sparks and Markesbery, 1991)	Loss of cholinergic neurons in nucleus basalis, selected midbrain nuclei and pedunculopontine nucleus	(Jellinger, 1988) (Kasashima and Oda, 2003) (Javoy-Agid, 1994) (Juncos *et al.*, 1991)	Loss of cholinergic neurons in nucleus basalis	(Kasashima and Oda, 2003)
			Pre synaptic cholinergic neurons reduced in the nucleus basalis, putamen, caudate and pallidum.	(Ruberg *et al.*, 1985) (Javoy-Agid, 1994) (Suzuki *et al.*, 2002) (Mazere *et al.*, 2012) (Shinotoh *et al.*, 1999) (Gilman *et al.*, 2010) (Hirano *et al.*, 2010)		
	No evidence of cholinergic neuron loss in the cerebral cortex	(Wood *et al.*, 1983) (Hirano *et al.*, 2010) (Procter *et al.*, 1999) (Hansen *et al.*, 1988)	Loss of cholinergic neurons in cerebral cortex	(Ruberg *et al.*, 1985) (Javoy-Agid, 1994) (Hirano *et al.*, 2010)	Reduced cholinergic neurons in the frontal, parietal and occipital cortex	(Hirano *et al.*, 2010)
	Conflicting results on cholinergic receptors	(Weinberger *et al.*, 1991) (Wood *et al.*, 1983) (Procter *et al.*, 1999)	No change in muscarinic receptor density	(Ruberg *et al.*, 1985) (Asahina *et al.*, 1998)		
	Atrophy of cholinergic basal nuclei in PPA	(Teipel *et al.*, 2016)				
**Glutamate**						
	Glutamatergic neurons	(Ferrer, 1999)	Glutamatergic neurons	(Henderson *et al.*,	No evidence	

	reduced in the thalamus, frontal and temporal cortex		reduced in the basal ganglia	2000)		
	Glutamate levels reduced in the frontal and temporal lobes	(Ernst *et al.*, 1997) (Sarac *et al.*, 2008)	NMDA receptors unchanged in the frontal and temporal lobes	(Holemans *et al.*, 1991)		
	AMPA and NMDA receptor levels reduced in the frontal and temporal lobes	(Francis *et al.*, 1993) (Procter *et al.*, 1999) (Bowen *et al.*, 2008)				
	Metabotropic glutamate receptors reduced in the cerebral cortex, basal ganglia and thalamus	(Leuzy *et al.*, 2016)				
**GABA**						
	GABAergic neurons reduced in the frontal and temporal lobes	(Ferrer, 1999)	GABAergic neurons reduced in the basal ganglia	(Levy *et al.*, 1995)	No evidence	
	GABA levels reduced in the basal ganglia	(Kanazawa *et al.*, 1988)	GABA receptors binding reduced	(Landwehrmeyer and Palacios, 1994) (Suzuki *et al.*, 2002) (Foster *et al.*, 2000)		

References

Albani D, Prato F, Fenoglio C, Batelli S, Dusi S, De Mauro S, et al. Association study to evaluate the serotonin transporter and apolipoprotein E genes in frontotemporal lobar degeneration in Italy. J. Hum. Genet. 2008; 53: 1029–1033.

Arnold G, Schwarz J, Tatsch K, Kraft E, Wächter T, Bandmann O, et al. Steele-Richardson-Olszewski-syndrome: The relation of dopamine D2 receptor binding and subcortical lesions in MRI. J. Neural Transm. 2002; 109: 503–512.

Asahina M, Suhara T, Shinotoh H, Inoue O, Suzuki K, Hattori T. Brain muscarinic receptors in progressive supranuclear palsy and Parkinson's disease: a positron emission tomographic study. J. Neurol. Neurosurg. Psychiatry 1998; 65: 155–63.

Baron JC, Maziere B, Loc'h C, Cambon H, Sgouropoulos P, Bonnet AM, et al. Loss of striatal [76Br]bromospiperone binding sites demonstrated by positron tomography in progressive supranuclear palsy. J Cereb Blood Flow Metab 1986; 6: 131–6.

Bowen DM, Procter AW, Mann DMA, Snowden JS, Esiri MM, Neary D, et al. Imbalance of a serotonergic system in frontotemporal dementia: Implication for pharmacotherapy. Psychopharmacology (Berl). 2008; 196: 603–610.

Brooks DJ, Ibanez V, Sawle G V, Playford ED, Quinn N, Mathias CJ, et al. Striatal D2 receptor status in patients with Parkinson's disease, striatonigral degeneration, and progressive supranuclear palsy, measured with 11C-raclopride and positron emission tomography. Ann. Neurol. 1992; 31: 184–92.

Brunnström H, Friberg N, Lindberg E, Englund E. Differential degeneration of the locus coeruleus in dementia subtypes. Clin. Neuropathol. 2011; 30: 104–10.

Castro ME, Pascual J, Romon T, Pazos A. 5-HT 1B receptor binding in degenerative movement disorders. Brain Res. 1998; 790: 323–328.

Chaal S, Rowe J. Dopamine Transporter (DAT) imaging can be normal with neuropathologically confirmed Cortiobasal Degeneration. J. Neurol. Neurosurg. Psychiatry 2013; 84: e2.59-e2.

Chinaclia G, Landwehrmeyer B. Serotoninergic Terminal Transporters Are Differentially Affected in Parkinson ' S Disease and Progressive Supranuclear Palsy : an Autoradiographic Study With [3H] Citalopram. Neuroscience 1993; 54: 691–699.

Cilia R, Rossi C, Frosini D, Volterrani D, Siri C, Pagni C, et al. Dopamine transporter spect imaging in corticobasal syndrome. PLoS One 2011; 6

Dickson DW. Neuropathologic differentiation of progressive supranuclear palsy and corticobasal degeneration. J. Neurol. 1999; 246 Suppl: II6-I15.

Dickson DW, Ahmed Z, Algom AA, Tsuboi Y, Josephs KA. Neuropathology of variants of progressive supranuclear palsy. Curr. Opin. Neurol. 2010; 23: 394–400.

Engelborghs S, Vloeberghs E, Le Bastard N, Van Buggenhout M, Mariën P, Somers N, et al. The dopaminergic neurotransmitter system is associated with aggression and agitation in frontotemporal dementia. Neurochem. Int. 2008; 52: 1052–1060.

Ernst T, Chang L, Melchor R, Mehringer CM. Frontotemporal dementia and early Alzheimer disease: differentiation with frontal lobe H-1 MR spectroscopy. Radiology 1997; 203: 829–836.

Ferrer I. Neurons and their dendrites in frontotemporal dementia. Dement. Geriatr. Cogn. Disord. 1999; 10: 55–60.

Foster NL, Minoshima S, Johanns J, Little R, Heumann ML, Kuhl DE, et al. PET measures of benzodiazepine receptors in progressive supranuclear palsy. Neurology 2000; 54: 1768–1773.

Franceschi M, Anchisi D, Pelati O, Zuffi M, Matarrese M, Moresco RM, et al. Glucose metabolism and serotonin receptors in the frontotemporal lobe degeneration. Ann. Neurol. 2005; 57: 216–225.

Francis PT, Holmes C, Webster MT, Stratmann GC, Procter AW, Bowen DM. Preliminary neurochemical findings in non-Alzheimer dementia due to lobar atrophy. Dementia 1993; 4: 172–177.

Frisoni GB, Pizzolato G, Bianchetti a, Chierichetti F, Ferlin G, Battistin L, et al. Single photon emission computed tomography with [99Tc]-HM-PAO and [123I]-IBZM in Alzheimer's disease and dementia of frontal type: preliminary results. Acta Neurol. Scand. 1994; 89: 199–203.

Gibb WRG, Luthert PJ, Marsden CD. Corticobasal Degeneration. Brain 1989; 112: 1171 LP-1192.

Gilman S, Koeppe RA, Nan B, Wang CN, Wang X, Junck L, et al. Cerebral cortical and subcortical cholinergic deficits in parkinsonian syndromes. Neurology 2010; 74: 1416–1423.

Gil-Navarro S, Lomeña F, Cot A, Lladó A, Montagut N, Castellví M, et al. Decreased striatal dopamine transporter uptake in the non-fluent/agrammatic variant of primary progressive aphasia. Eur. J. Neurol. 2013; 20: 1459–1466.

Hansen LA, Deteresa R, Tobias H, Alford M, Terry RD. Neocortical morphometry and cholinergic neurochemistry in Pick's disease. Am. J. Pathol. 1988; 131: 507–18.

Hardman CD, Halliday GM, McRitchie D a, Cartwright HR, Morris JG. Progressive supranuclear palsy affects both the substantia nigra pars compacta and reticulata. Exp. Neurol. 1997; 144: 183–192.

Hauw J, Daniel SE, Dickson D, Horoupian DS. Preliminary NINDS neuropathologic criteria for Steele-Richardson-Olszewski syndrome (progressive supranuclear palsy). Neurology. 1994; 44: 2015–2019.

Henderson JM, Carpenter K, Cartwright H, Halliday GM. Loss of thalamic intralaminar nuclei in progressive supranuclear palsy and Parkinson's disease: clinical and therapeutic implications. Brain 2000; 123 (Pt 7: 1410–1421.

Hirano S, Shinotoh H, Shimada H, Aotsuka A, Tanaka N, Ota T, et al. Cholinergic imaging in corticobasal syndrome, progressive supranuclear palsy and frontotemporal dementia. Brain 2010; 133: 2058–2068.

Holemans S, Javoy F, Agid Y, Laterre EC, Maloteaux JM. [3H]MK-801 binding to NMDA glutamatergic receptors in Parkinson's disease and progressive supranuclear palsy. Brain Res. 1991; 565: 154–157.

Hornykiewicz O, Shannak K. Brain monoamines in progressive supranuclear palsy--comparison with idiopathic Parkinson's disease. J. Neural Transm. Suppl. 1994; 42: 219–227.

Im J-H, Chung SJ, Kim J-S, Lee MC. Differential patterns of dopamine transporter loss in the basal ganglia of progressive supranuclear palsy and Parkinson's disease: analysis with [(123)I]IPT single photon emission computed tomography. J. Neurol. Sci. 2006; 244: 103–109.

Irwin DJ, Brettschneider J, McMillan CT, Cooper F, Olm C, Arnold SE, et al. Deep clinical and neuropathological phenotyping of Pick disease. Ann. Neurol. 2016; 79: 272–287.

Javoy-Agid F. Cholinergic and peptidergic systems in PSP. J. Neural Transm. Suppl. 1994; 42: 205–218.

Jellinger K. The pedunculopontine nucleus in Parkinson's disease, progressive supranuclear palsy and Alzheimer's disease. J. Neurol. Neurosurg. Psychiatry 1988; 51: 540–3.

Juncos JL, Hirsch EC, Malessa S, Duyckaerts C, Hersh LB, Agid Y. Mesencephalic cholinergic nuclei in progressive supranuclear palsy. Neurol. VO - 41 1991; 289: 25.

Kaasinen V, Gardberg M, Röyttä M, Seppänen M, Päivärinta M. Normal dopamine transporter SPECT in neuropathologically confirmed corticobasal degeneration. J. Neurol. 2013; 260: 1410–1411.

Kanazawa I, Kwak S, Sasaki H, Muramoto O, Mizutani T, Hori A, et al. Studies on neurotransmitter markers of the basal ganglia in Pick's disease, with special reference to dopamine reduction. J. Neurol. Sci. 1988; 83: 63–74.

Kasashima S, Oda Y. Cholinergic neuronal loss in the basal forebrain and mesopontine tegmentum of progressive supranuclear palsy and corticobasal degeneration. Acta

Neuropathol. 2003; 105: 117–24.

Klaffke S, Kuhn AA, Plotkin M, Amthauer H, Harnack D, Felix R, et al. Dopamine transporters, D2 receptors, and glucose metabolism in corticobasal degeneration. Mov. Disord. 2006; 21: 1724–1727.

Lanctôt KL, Herrmann N, Ganjavi H, Black SE, Rusjan PM, Houle S, et al. Serotonin-1A receptors in frontotemporal dementia compared with controls. Psychiatry Res. - Neuroimaging 2007; 156: 247–250.

Landwehrmeyer B, Palacios JM. Alterations of neurotransmitter receptors and neurotransmitter transporters in progressive supranuclear palsy. J. Neural Transm. Suppl. 1994; 42: 229–246.

Laureys S, Salmon E, Garraux G, Peigneux P, Lemaire C, Degueldre C, et al. Fluorodopa uptake and glucose metabolism in early stages of corticobasal degeneration. J. Neurol. 1999; 246: 1151–1158.

Leuzy A, Zimmer ER, Dubois J, Pruessner J, Cooperman C, Soucy JP, et al. In vivo characterization of metabotropic glutamate receptor type 5 abnormalities in behavioral variant FTD. Brain Struct. Funct. 2016; 221: 1387–1402.

Levy R, Ruberg M, Herrero MT, Villares J, Javoy-Agid F, Agid Y, et al. Alterations of GABAergic neurons in the basal ganglia of patients with progressive supranuclear palsy: an in situ hybridization study of GAD67 messenger RNA. Neurology 1995; 45: 127–134.

Mazere J, Meissner WG, Mayo W, Sibon I, Lamare F, Guilloteau D, et al. Progressive supranuclear palsy: in vivo SPECT imaging of presynaptic vesicular acetylcholine transporter with [123I]-iodobenzovesamicol. Radiology 2012; 265: 537–543.

Mori H, Oda M, Komori T, Arai N, Takanashi M, Mizutani T, et al. Lewy bodies in progressive supranuclear palsy. Acta Neuropathol. 2002; 104: 273–278.

Murphy KE, Karaconji T, Hardman CD, Halliday GM. Excessive dopamine neuron loss in progressive supranuclear palsy. Mov. Disord. 2008; 23: 607–610.

Nagaoka S, Arai H, Iwamoto N, Ohwada J, Ichimiya Y, Nakamura M, et al. A juvenile case of frontotemporal dementia: Neurochemical and neuropathological investigations. Prog. Neuropsychopharmacol. Biol. Psychiatry 1995; 19: 1251–1261.

Nagasawa H, Tanji H, Nomura H, Saito H, Itoyama Y, Kimura I, et al. PET study of cerebral glucose metabolism and fluorodopa uptake in patients with corticobasal degeneration. J. Neurol. Sci. 1996; 139: 210–7.

Oh M, Kim JS, Kim JY, Shin K-H, Park SH, Kim HO, et al. Subregional Patterns of Preferential Striatal Dopamine Transporter Loss Differ in Parkinson Disease, Progressive Supranuclear Palsy, and Multiple-System Atrophy. J. Nucl. Med. 2012; 53: 399–406.

Oyanagi C. Comparison of Striatal Dopamine D2 Receptors in Parkinson ' s Disease and Progressive Supranuclear Palsy Patients Using [I] Iodobenzofuran Single-Photon Emission Computed Tomography. J. Neuroimaging 2002; 12: 316–324.

Oyanagi K, Tsuchiya K, Yamazaki M, Ikeda K. Substantia nigra in progressive supranuclear palsy, corticobasal degeneration, and parkinsonism-dementia complex of Guam: specific pathological features. J. Neuropathol. Exp. Neurol. 2001; 60: 393–402.

Pal PK, Wszolek ZK, Kishore A, De La Fuente-Fernandez R, Sossi V, Uitti RJ, et al. Positron emission tomography in pallido-ponto-nigral degeneration (PPND) family (frontotemporal dementia with parkinsonism linked to chromosome 17 and point mutation in tau gene). Park. Relat. Disord. 2001; 7: 81–88.

Pascual J, Berciano J, Grijalba B, del OE, Gonzalez AM, Figols J, et al. Dopamine D1 and D2 receptors in progressive supranuclear palsy: an autoradiographic study. Ann. Neurol 1992; 32: 703–707.

Pierot L, Desnos C, Blin J, Pierrot L, Scherman D, Javoy-agid F, et al. D1 and D2-type dopamine receptors in patients with Parkinson's disease and progressive supranuclear palsy. J. … 1988; 86: 291–306.

Pirker S, Perju-Dumbrava L, Kovacs GG, Traub-Weidinger T, Asenbaum S, Pirker W. Dopamine D2 receptor SPECT in corticobasal syndrome and autopsy-confirmed corticobasal degeneration. Park. Relat. Disord. 2013; 19: 222–226.

Pirker S, Perju-Dumbrava L, Kovacs GG, Traub-Weidinger T, Pirker W. Progressive dopamine transporter binding loss in autopsy-confirmed Corticobasal Degeneration. J. Parkinsons. Dis. 2015; 5: 907–912.

Premi E, Archetti S, Pilotto A, Seripa D, Paghera B, Padovani A, et al. Functional genetic variation in the serotonin 5-HTTLPR modulates brain damage in frontotemporal dementia. Neurobiol. Aging 2015; 36: 446–451.

Procter AW, Qurne M, Francis PT. Neurochemical features of frontotemporal dementia. Dement. Geriatr. Cogn. Disord. 1999; 10: 80–84.

Revesz T, Sangha H, Daniel SE. The nucleus raphe interpositus in the Steele-Richardson-Olszewski syndrome (progressive supranuclear palsy). Brain 1996; 119 (Pt 4: 1137–1143.

Rinne JO, Laine M, Kaasinen V, Norvasuo-Heila MK, Nagren K, Helenius H. Striatal dopamine transporter and extrapyramidal symptoms in frontotemporal dementia. Neurology 2002; 58: 1489–1493.

Ruberg M, Javoy-Agid F, Hirsch E, Scatton B, LHeureux R, Hauw JJ, et al. Dopaminergic and cholinergic lesions in progressive supranuclear palsy. Ann. Neurol. 1985; 18: 523–529.

Sarac H, Zagar M, Davorka V, Henigsberg Ne, Bilic E, Pavlisa G. Magnetic Resonance Imaging and Magnetic Resonance Spectroscopy in a Patient with Amyotrophic Lateral Sclerosis and Frontotemporal Dementia. Coll. Antropol 2008; 32: 205–210.

Sawle G V, Brooks DJ, Marsden CD, Frackowiak RSJ. Corticobasal degeneration - A unique pattern of regional cortical oxygen hypometabolism and striatal fluorodopa uptake demonstrated by positron emmission tomography. Brain 1991; 114: 541 LP-556.

Sedaghat F, Gotzamani-Psarrakou A, Dedousi E, Arnaoutoglou M, Psarrakos K, Baloyannis I, et al. Evaluation of dopaminergic function in frontotemporal dementia using I-FP-CIT single photon emission computed tomography. Neurodegener. Dis. 2007; 4: 382–5.

Shinotoh H, Namba H, Yamaguchi M, Fukushi K, Nagatsuka SI, Iyo M, et al. Positron emission tomographic measurement of acetylcholinesterase activity reveals differential loss of ascending cholinergic systems in Parkinson’s disease and progressive supranuclear palsy. Ann. Neurol. 1999; 46: 62–69.

Sjogren M, Minthon L, Passant U, Blennow K, Wallin A. Decreased monoamine metabolites in frontotemporal dementia and Alzheimer’s disease. Neurobiol. Aging 1998; 19: 379–384.

Sparks DL, Markesbery WR. Altered serotonergic and cholinergic synaptic markers in Pick’s disease. Arch. Neurol. 1991; 48: 796–799.

Stamelou M, Matusch A, Elmenhorst D, Hurlemann R, Eggert KM, Zilles K, et al. Nigrostriatal upregulation of 5-HT2A receptors correlates with motor dysfunction in progressive supranuclear palsy. Mov. Disord. 2009; 24: 1170–1175.

Suzuki M, Desmond TJ, Albin RL, Frey K a. Cholinergic vesicular transporters in progressive supranuclear palsy. Neurology 2002; 58: 1013–8.

Teipel S, Raiser T, Riedl L, Riederer I, Schroeter ML, Bisenius S, et al. Atrophy and structural covariance of the cholinergic basal forebrain in primary progressive aphasia. Cortex 2016; 83: 124–135.

Vermeiren Y, Le Bastard N, Van Hemelrijck A, Drinkenburg WH, Engelborghs S, De Deyn PP. Behavioral correlates of cerebrospinal fluid amino acid and biogenic amine neurotransmitter alterations in dementia. Alzheimer’s Dement. 2013; 9: 488–498.

Vermeiren Y, Janssens J, Aerts T, Martin J-J, Sieben A, Van Dam D, et al. Brain Serotonergic and Noradrenergic Deficiencies in Behavioral Variant Frontotemporal Dementia Compared to Early-Onset Alzheimer’s Disease. J. Alzheimer’s Dis. 2016; Preprint: 1–18.

Warren NM, Piggott MA, Lees AJ, Burn DJ. The basal ganglia cholinergic neurochemistry of progressive supranuclear palsy and other neurodegenerative diseases. J Neurol Neurosurg Psychiatry 2007; 78: 571–575.

Weinberger DR, Gibson R, Coppola R, Jones DW, Molchan S, Sunderland T, et al. The distribution of cerebral muscarinic acetylcholine receptors in vivo in patients with dementia. A controlled study with 123IQNB and single photon emission computed tomography. Arch. Neurol. 1991; 48: 169–76.

Wood PL, Etienne P, Lal S, Nair NP, Finlayson MH, Gauthier S, et al. A post-mortem comparison of the cortical cholinergic system in Alzheimer's disease and Pick's disease. J. Neurol. Sci. 1983; 62: 211–217.

Yang Y, Schmitt HP. Frontotemporal dementia: evidence for impairment of ascending serotoninergic but not noradrenergic innervation. Immunocytochemical and quantitative study using a graph method. Acta Neuropathol 2001; 101: 256–270.

Yokoyama JS, Bonham LW, Sturm VE, Adhimoolam B, Karydas A, Coppola G, et al. The 5-HTTLPR variant in the serotonin transporter gene modifies degeneration of brain regions important for emotion in behavioral variant frontotemporal dementia Serotonin transporter in bvFTD. NeuroImage Clin. 2015; 9: 283–290.
